# Functionalization of Rhodamine Platforms with 3-Hydroxy-4-pyridinone Chelating Units and Its Fluorescence Behavior towards Fe(III)

**DOI:** 10.3390/molecules27051567

**Published:** 2022-02-26

**Authors:** Carla Queirós, Sílvia Vinhas, Jéssica Oliveira, Andreia Leite, Ana M. G. Silva, Maria Rangel

**Affiliations:** 1 LAQV/REQUIMTE, Departamento de Química e Bioquímica, Faculdade de Ciências da Universidade do Porto, 4169-007 Porto, Portugal; carla.queiros@fc.up.pt (C.Q.); up201002657@edu.fc.up.pt (S.V.); jessicafernandar392@gmail.com (J.O.); 2 Departamento de Ciências Exatas, Universidade Estadual de Feira de Santana (UEFS), Avenida Transnordestina, Novo Horizonte 44036-900, BA, Brazil; 3 LAQV/REQUIMTE, Instituto de Ciências Biomédicas de Abel Salazar, 4099-003 Porto, Portugal; mrangel@icbas.up.pt

**Keywords:** dihydrorosamine, rhodamine 110, pyridinone units, amide coupling, optical properties, fluorescence

## Abstract

Functionalization of xanthene fluorophores with specific receptor units is an important topic of research aiming for the development of new analytical tools for biological sciences, clinical diagnosis, food and environmental monitoring. Herein, we report a new dihydrorosamine containing two active amino groups, which was functionalized with 3-benzyloxy-1-(3′-carboxypropyl)-2-methyl-4-pyridinone through an amide coupling strategy. Benzylated mono- and di-functionalized dihydrorosamine derivatives (H in position 9 of the xanthene) were obtained, but with modest reaction yields, requiring long and laborious purification procedures. Looking for a more efficient approach, rhodamine 110 was selected to react with the carboxypropyl pyridinone, enabling the isolation of the corresponding mono- and di-functionalized derivatives in amounts that depend on the excess of pyridinone added to the reaction. The structure of all compounds was established by ^1^H and ^13^C NMR, MS (ESI) and their absorption and emission properties were evaluated in dichloromethane. The fluorescence behavior of the debenzylated mono-rhodamine 110 derivative in the presence of Fe(III) was studied, making it an interesting fluorogenic dye for future optical sensing applications.

## 1. Introduction

**Figure 1:**
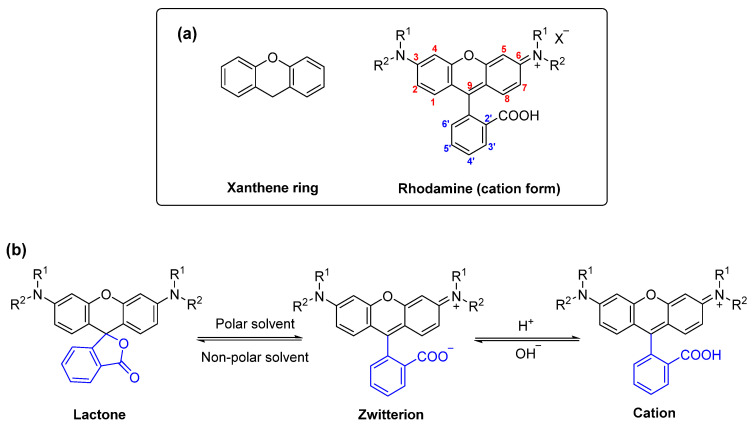
(**a**) General structure of xanthene and rhodamine dyes; (**b**) Molecular structures of three rhodamine forms in equilibrium.

Rhodamines are a family of fluorescent dyes based on a planar aromatic xanthene core, whose general structures are represented in Figure 1a. Due to their excellent photophysical properties in solution, particularly high molar absorptivity, intense fluorescence spectrum in the visible region, high quantum yield and photostability, rhodamines continue to be excellent scaffolds to be used as fluorescent probes and their applications range from chemistry and biology to materials science [1,2]. For example, rhodamine spirolactams incorporating nitrogen donors have been applied as photochemical switching systems for the selective colorimetric detection of metal ions in an aqueous solution and living cells [3]. Other examples include their use as thermometers [4,5], fluorescent probes in sensing various biological species [2,6], as scaffolds in biological labeling and *turn-on* fluorescence imaging [7] and as antibacterial, antiviral, anti-inflammatory and antitumor agents in biological applications [8,9], among others.

In a solution, the photophysical properties of rhodamines are dependent on the molecular structure of the dye, as well as, environment effects, including solvent, pH and temperature [10]. The equilibrium between “closed” spirolactone and “open” quinoid forms controls the conjugation of the rings (Figure 1b). In non-polar solvents, rhodamines exist in the spirolactone form; thus, their molar extinction coefficient (and also fluorescence quantum yield and lifetime) are very low, on account of the interruption of π-conjugation of the xanthene core. However, the lactone form undergoes the opening of the ring into a zwitterionic form in polar solvents, or a cationic form in acidic media, recovering the color and fluorescence.

Rosamines are rhodamine analogues that lack the carboxylic acid group at the *ortho* position of the phenyl ring, and therefore are unable to participate in spirolactone-quinoid equilibria. Rosamines are often considered advantageous over rhodamines as they exhibit very attractive photophysical properties and are usually easier to synthesize and to purify [11,12,13,14].

From the wide variety of chemical modifications available on rhodamines [6], one of the most relevant involves the modification of the two reactive amino groups of the xanthene moiety (positions 3 and 6). Amongst others, Rhodamine 110 (**Rho110**) is characterized by an absorption maximum at 497 nm, emission maximum at 520 nm, and a high quantum yield of 0.85 in water [7]. This dye contains both unsubstituted amino groups that can be modified either by reaction with an acyl chloride (or chloroformate) or with a carboxylic acid using a carbodiimide as a coupling agent [1]. Both mono- and di-functionalized derivatives of **Rho110** can be achieved using these procedures.

The exceptional photophysical properties of rhodamines can be complemented with the introduction of receptors, such as 3-hydroxy-4-pyridinone (3,4-HPO) units, allowing the optical detection of several species, namely M(II) and M(III) metal ions [15]. Indeed, 3,4-HPOs are an important class of *N*-heterocyclic bidentate ligands, whose use has been widely explored as specific and selective chelators towards environmentally and biologically relevant metals, specially Al^3+^ and Fe^3+^ [16,17,18,19]. In this context, the functionalization at the benzoate ring of rhodamine with 3,4-HPO chelating units is being approached as a very promising and useful methodology to obtain fluorescent probes, which can be used in the detection and quantification of such metal ions [15,16,17,18,19]. 

In this work, we are exploring the functionalization of two rhodamine dyes (dihydrorosamine **2** and **Rho110**) at the nitrogen positions of the xanthene structure, with 3-benzyloxy-1-(3′-carboxypropyl)-2-methyl-4-pyridinone (**L^1^**), in order to obtain mono- and di-functionalized derivatives. The structure of the resulting derivatives was established by ^1^H and ^13^C NMR, MS (ESI) and their optical (absorption and emission) properties were evaluated, being the mono-functionalized derivative debenzylated and used in fluorescence studies in the presence of Fe(III) metal ion.

## 2. Results and Discussion

### 2.1. Synthesis

Rosamine **1** was initially prepared from the condensation of 3-aminophenol with benzaldehyde using propionic acid and a catalytic amount of *p*-toluenesulfonic acid at 60–80 °C, followed by oxidation with 2,3-dichloro-5,6-dicyano-1,4-benzoquinone (DDQ), to give **3** in 14% isolated yield. Better results were obtained by performing the condensation in methanosulfonic acid at 150 °C during 16 h, which allowed the isolation of **1** in 50% of yield. In this case, the oxidative cyclization occurred in situ, meaning that no extra oxidative step was necessary to complete the rosamine scaffold.

**Scheme 1:**
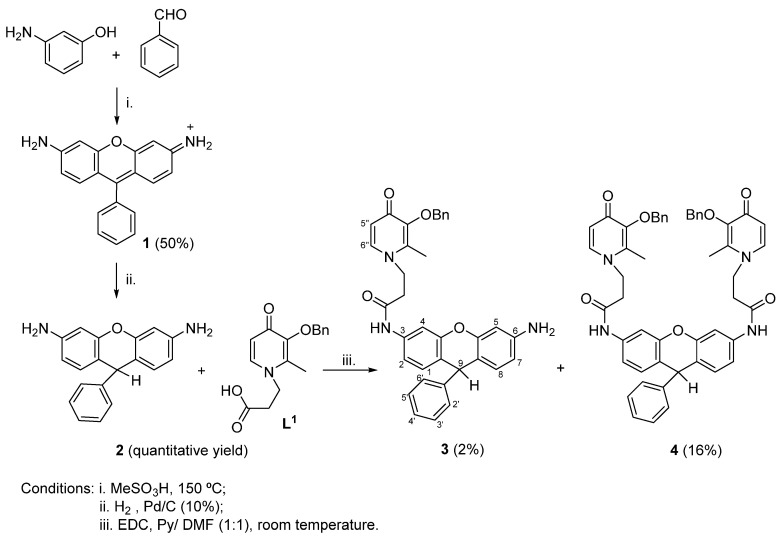
Synthesis of rosamine **1**, followed by catalytic hydrogenation to give dihydrorosamine **2**, and subsequent amide coupling of **2** and **L^1^** to yield mono- and di-functionalized derivatives **3** and **4**.

Next, we tested the amide coupling reaction of rosamine **1** with the carboxypropyl pyridinone **L^1^**, but no reaction was observed. We assumed that through the reduction of the xanthene core the reactivity of the nitrogen atoms at positions 3 and 6 of the xanthene would increase, allowing the coupling with carboxylic acids using mild conditions. Accordingly, we performed the reduction of rosamine **1** by catalytic hydrogenation (H_2_, 10% Pd/C), to give quantitatively the dihydrorosamine **2**. Subsequently, the reaction of **2** with the carboxypropyl pyridinone **L^1^** (2.6 equiv.) using *N-*(3-dimethylaminopropyl)-*N*′-ethylcarbodiimide hydrochloride (EDC) in a 1:1 mixture of anhydrous pyridine (Py) and DMF [20] furnished the mono- and di-functionalized derivatives **3** and **4** in 2% and 16% of yield, respectively (Scheme 1). It is important to mention that, in addition to the desired conjugates **3** and **4**, the control by TLC analysis of the reaction mixture revealed the presence of other compounds, including (i) starting unreacted dihydrorosamine **2**, (ii) rosamine **1** probably regenerated during the reaction and/or during the TLC plate elution through contact with the silica gel used as a stationary phase, and (iii) other minor by-products. These observations revealed that the reaction conversion is not complete, and that the compounds obtained are unstable (tendency to oxidize), which explains the low yields obtained by this approach.

**Scheme 2:**
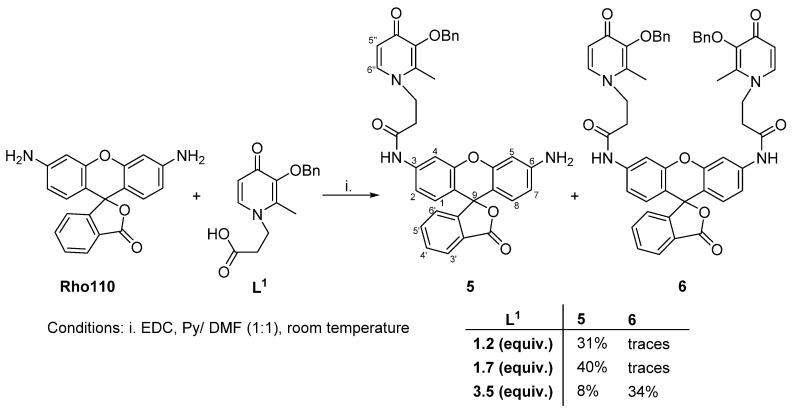
Amide coupling of **Rho110** and **L^1^** to give mono- and di-functionalized derivatives **5** and **6**.

In order to find a more efficient approach, we decided to replace dihydrorosamine **2** by the commercially available Rhodamine 110 (**Rho110**). **Rho110** contains two active amino groups, the *ortho*-carboxyl group in the phenyl ring and can exist in equilibrium of the “open” quinoid and “closed” lactone forms, being the “closed” lactone form typically more reactive for synthetic proposes. The condensation of **Rho110** with 1.2 equiv. of **L^1^**, using EDC in Py/DMF (1:1) was achieved at room temperature for 24 h (Scheme 2). After the preparative thin-layer chromatographic purification, the mono-functionalized derivative **5** was isolated in 31% of yield. In addition, 55% of **Rho110** was recovered unchanged, along with traces of the di-functionalized derivative **6**. The use of a bigger amount of **L^1^** (1.7 equiv.) led to the formation of **5** in 40% of the yield, while only traces of **6** were detected. Finally, when the reaction was carried out with a significant excess of **L^1^** (3.5 equiv.), conjugates **5** and **6** were isolated in 8% and 34% yields, respectively. Note that in all these cases, the mono- and di-functionalized derivatives were isolated mainly in “closed” lactone.

Recently, Jing Shi and coworkers [21] have successfully performed the condensation reaction of **Rho110** and Boc glycine in DMF using 1-[bis(dimethylamino)methylene]-1H-1,2,3-triazolo[4,5-b]pyridinium 3-oxid hexafluorophosphate (HATU) in the presence of *N*,*N*-diisopropylethylamine (DIPEA). We have tried similar conditions, involving the initial treatment of **L^1^** (4 equiv.) with HATU (3.5 equiv.) in a mixture of DMF and DIPEA (18 equiv.), with subsequent addition of **Rho110** (1 equiv.). Unfortunately, these conditions led to reactions with modest yields (monosubstituted in 21% yield and disubstituted in very low yield) together with many difficulties in the purification process.

The condensation through the formation of acyl chloride from **L^1^** was also attempted. Adding thionyl chloride (5 equiv.) and DIPEA to a solution of **L^1^** (3 equiv.) in DMF (18 h at room temperature), resulted in the formation of monosubstituted derivative **5** in 24% of the yield.

Considering the objective of Fe(III) sensing and the stoichiometric of the corresponding Fe-chelates, we selected the bidentate derivative (**5**) for further studies bearing in mind the advantage of leaving an extra position for a different functional group. In fact, previous results of synthetic work on rigid structures with two 3,4-HPO arms showed that a planar linker favors the use of the ligand as a bidentate rather than a tetradentate one [22].

**Scheme 3:**
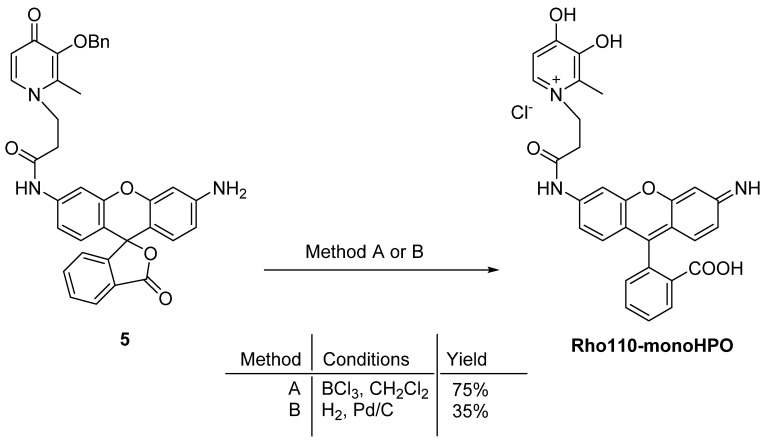
Deprotection of the benzyl ether of mono-functionalized derivative **5** using BCl_3_ in CH_2_Cl_2_ (Method A) or H_2_ in the presence of Pd/C (Method B).

Therefore, we decided to proceed with the deprotection of the benzyl group for derivative **5**, using the Lewis acid boron trichloride (BCl_3_), which allowed the isolation of the desired hydrochloride salt of **Rho110-monoHPO** in 75% of the yield (Scheme 3, Method A). Being milder and less acidic, the treatment under hydrogen atmosphere over 10% Pd/C was also attempted to deprotect **5**. An orange powder was isolated, which after ^1^H NMR analysis revealed to be the deprotected compound in the reduced form (H insertion in position 9 of the xanthene). Keeping this derivative in the solution at room temperature for 24 h, we found that it can be totally converted into the oxidized form, without any further treatment, affording **Rho110-monoHPO** in 35% of the yield (Scheme 3, Method B).

### 2.2. NMR Characterization

^1^H NMR, ^13^C NMR and two-dimensional NMR experiments, including gHSQC and gHMBC, for carbon assignment are provided in Supplementary Materials. Figure S25 of Supplementary Materials shows a comparison of the ^1^H NMR spectra of the synthesized rosamine **1** and dihydrorosamine **2**, where the appearance of an additional singlet at 5.48 ppm corresponding to the resonance of 9-H proton in derivative **2** can be observed.

The coupling reaction of dihydrorosamine **2** with the pyridinone afforded the mono- and di-functionalized derivatives **3** and **4**, whose comparison of the ^1^H NMR spectra is presented in Figure S26 in Supplementary Materials. Both spectra show the two characteristic doublets at 7.73 ppm and 6.45–6.48 ppm, due to H-6″ and H-5″, respectively, and the singlet at 5.19–5.20 ppm due to H-9. Differences in chemical shifts are perceptible for the xanthene and phenyl protons attributed mainly to the asymmetric nature of **3**, which contrasts with the high symmetry of **4**. Additionally, in ^1^H NMR spectra of compounds **5** and **6** (Figure S27 in Supplementary Materials), signals due to H-6″ and H-5″ of the pyridinone remain identical in both compounds.

The ^1^H NMR spectrum of the isolated **Rho110-monoHPO** (Figure S23 in Supplementary Materials) shows the absence of signals due to the benzyl protecting group and a significant deshielding of the two doublets at 8.31 and 7.21 ppm, due to the H-6″ and H-5″ of the pyridinone, which confirms that the ligand is obtained in the dihydroxypyridinium form.

The HRMS (ESI) spectra (provided in Supplementary Materials) confirmed the structures proposed, exhibiting the molecular ion (M+H^+^) of compounds **3** and **4** at *m/z* values 558.24 and 827.34, respectively, of compounds **5** and **6** at *m/z* values 600.22 and 869.32, respectively, and the (M^+•^) of **Rho110-monoHPO** at *m/z* value 510.17.

### 2.3. Spectroscopic Characterization

The spectroscopic characterization by UV-Vis and fluorescence studies, of the compounds was performed in solution using dichloromethane (CH_2_Cl_2_). Dichloromethane was chosen as a solvent as it can enhance the optical properties of rhodamine derivatives, namely the fluorescence quantum yield [12,23]. The results obtained for the compounds are summarized in Table 1, giving an insight into some spectroscopic differences between the rosamine and rhodamine derivatives.

Compounds **1** (*λ*_abs_/*λ*_em_ = 511 nm/526 nm) and **2** (*λ*_abs_/*λ*_em_ = 507 nm/530 nm) exhibit a bathochromic shift in absorption and emission wavelengths relative to **Rho110** (*λ*_abs_/*λ*_em_ = 498 nm/515 nm) (Figure S35 in Supplementary Materials). In contrast, derivatives **3** and **4** exhibit an absorption band at 274 and 270 nm, respectively, and lack of emission (Figure S35 in Supplementary Materials). This can be explained by the presence of the hydrogen atom at position 9 in the xanthene core which causes the loss of the aromaticity—a similar effect to the lactone form of the rhodamine derivatives.

**Figure 2:**
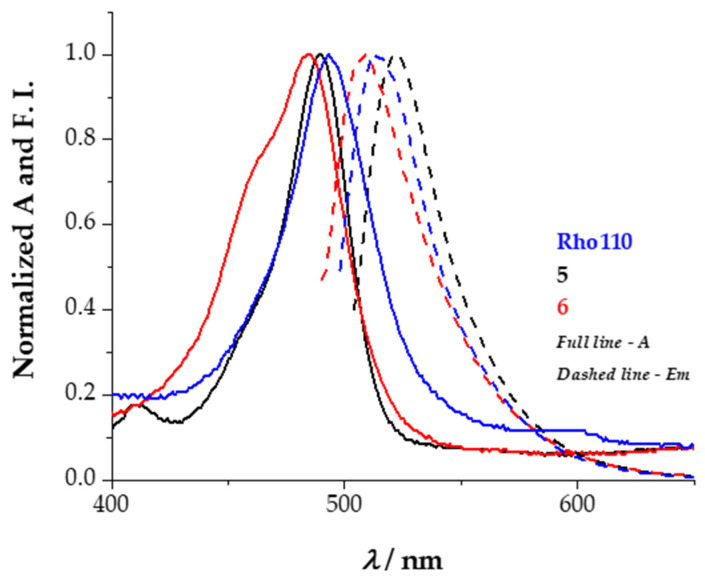
Normalized absorption and emission spectra of **Rho110** (blue), **5** (black, quinoid form) and **6** (red, quinoid form) in CH_2_Cl_2_ at 25 °C (full line—absorption spectra; dashed line—emission spectra).

Similarly, when compounds **5** and **6** are present in spirolactone forms, they are colorless, and that fact is reflected in the UV-Vis spectrum where the characteristic absorption band of rhodamine is absent (Figure S36 in Supplementary Materials). Only one band is observed at 270 nm (**5**) and 262 nm (**6**) assigned to the pyridinone moiety (Table 1). In order to convert them into the quinoid form [24,25], a small amount of HCl (37%) was added to a solution containing **5** or **6**. The spectra were acquired (Figure 2) and two major changes were observed: (i) a change in coloration from colorless to orange with appearance of a new absorption band at 489 nm (**5**) and 485 nm (**6**), and (ii) the appearance of an emission band at 521 nm (**5**) and 508 nm (**6**). These changes indicate that the acidic pH led to the opening of the lactone to the quinoid form. Comparing with **Rho110**, a hypsochromic shift of 9 and 13 nm was observed in the absorption band for quinoid forms **5** and **6,** respectively, while the emission bands follow different patterns—bathochromic shift for **5** (6 nm) and hypsochromic shift for **6** (7 nm). Additionally, the difference between *λ*_abs_ and *λ*_em_ (Stokes shift) is larger for the asymmetric mono-functionalized derivative **5** (Δ*λ* = 32 nm), which means that this derivative is less susceptible to self-quenching via energy transfer mechanisms and potentially useful for biological applications.

**Figure 3:**
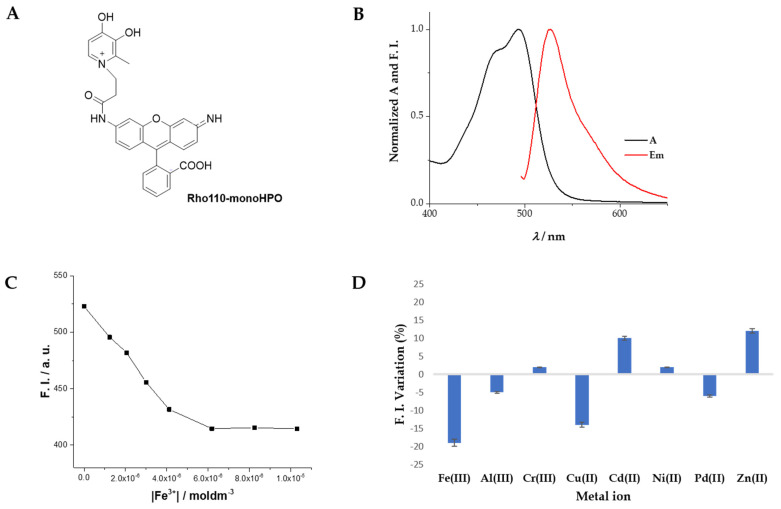
(**A**) Structure of the hydrochloride salt of **Rho110-monoHPO**; (**B**) Normalized absorption and emission spectra in MOPS pH = 7.40 of **Rho110-monoHPO**, at 25 °C; (**C**) Fluorescence decrease with increasing Fe(III) concentrations (aqueous solution, pH = 5), [**Rho110-monoHPO**] = 1.0 × 10^−5^ M; (**D**) Fluorescence intensity variation (%) for the metal ions studied (Fe(III), Al(III), Cr(III), Cu(II), Cd(II), Ni(II), Pd(II), and Zn(II) with **Rho110-monoHPO**, in deionized water (pH = 5) at 25 °C [**Rho110-monoHPO**] = 1.0 × 10^−5^ M; 1:1 (**Rho110-monoHPO**:metal ion).

Considering the lack of emission of **3** and **4** and the interesting properties of **5**, we decided to proceed with this derivative, by removing the benzyl protective group of the HPO unit, to obtain **Rho110-monoHPO** (Figure 3A).

From a ligand design perspective, **Rho110-monoHPO** comprises: (i) a 3,4-HPO unit that has high stability constants towards M(II)/M(III), and (ii) an amino group that opens the possibility of extra functionalization, which may allow, for example, an easy and stable incorporation of this compound into sensing materials for measuring purposes. As depicted in Figure 3B, **Rho110-monoHPO** exhibits two absorption bands at 489 and 507 nm in CH_2_Cl_2_, with absorption coefficients 4 times higher than the benzylated **5** in the quinoid form (*ε* = 4.3 × 10^3^ and 4.6 × 10^3^ M^−1^cm^−1^, respectively). In MOPs buffer (pH = 7.40), **Rho110-monoHPO** exhibits two similar absorption bands at 470 and 494 nm, with *ε* = 9.8 × 10^3^ and 11.4 × 10^3^ M^−1^cm^−1^, respectively, with the corresponding emission band at 527 nm.

The response of **Rho110-monoHPO** towards metal ions was accessed in deionized water (pH = 5), using several metal ions including Al(III), Cr(III), Fe(III), Cd(II), Cu(II), Ni(II), Pd(II), and Zn(II). **Rho110-monoHPO** presented higher sensitivity towards Fe(III) and Cu(II). A decrease in absorbance and emission intensities was observed with increasing amounts of the metal ion; the decreases where more pronounced for Fe(III). Figure 3C illustrates the variation in the fluorescence intensity of **Rho110-monoHPO** in the presence of increasing amounts of Fe(III). The observed fluorescence decrease (*ca* 19%) indicates the formation of the **Rho110-monoHPO** and Fe(III) complex, as expected for a bidentate ligand [15,26]. In Figure 3D, a comparison plot of the fluorescence intensity variation (%) of **Rho110-monoHPO** in the presence of the studied metal ions (1:1 ratio) is presented. From the analysis of Figure, we can infer that there is a higher sensitivity of **Rho110-monoHPO** towards Fe(III), even comparing with Cu(II).

Regarding the stability of complexes formed, several studies have been published where the stability constants of hydroxypyridinones, such as 3-hydroxy-4-pyridinones, with Fe(III) and Cu(II) have been determined and the values follow the Irving–Williams series [27]. In general, the overall log *β*_3_ for Fe(III) is between 35–39 [28,29] and the log *β*_2_ for Cu(II) is around 17–19 [30], being similar values expected for **Rho110-monoHPO**.

## 3. Materials and Methods

Reagents and solvents were purchased as reagent grade and used without further purification unless otherwise stated. Rhodamine 110 chloride (**Rho110**) was purchased from Aldrich.

NMR spectra were recorded with Bruker Avance III 400 spectrometer (400.15 MHz for ^1^H and 100.63 MHz for ^13^C). For compound **6a**, the ^13^C NMR spectrum was recorded on a Bruker Avance III HD 600 spectrometer operated at 150.92 MHz and equipped with pulse-gradient units capable of producing magnetic-field pulsed gradients in the z direction of 6.57 Gcm^−1^. Two-dimensional gradient selected ^1^H/^13^C heteronuclear single quantum coherence (gHSQC), and ^1^H/^13^C heteronuclear multiple bond coherence (gHMBC) spectra were acquired using the standard Bruker software (Bruker BioSpin GmbH, Rheinstetten, Germany). Chemical shifts (*δ*) are reported in ppm and coupling constants (*J*) in Hz; internal standard was TMS. High resolution MS analysis was carried out by electrospray ionization (ESI) in an LTQ-Orbitrap-XL instrument (Thermo Scientific, Waltham, MA, USA) with the following ESI source parameters: electrospray needle voltage 3 kV, sheath gas nitrogen 5, capillary temperature 275 °C, capillary voltage 37 V, and tube lens voltage 120 V. Ionization polarity was adjusted according to the sample.

Flash chromatography was carried out using silica gel (Merck, 230–400 mesh). Preparative thin-layer chromatography was carried out on 20 × 20 cm glass plates coated with Merck 60 silica gel (1 mm thick). Analytical TLC was carried out on precoated sheets with silica gel (Merck 60, 0.2 mm thick; Kenilworth, NJ, USA).

Electronic absorption spectra were recorded on a Shimadzu–UV 3600 UV–Vis-NIR equipped with a Shimadzu TCC-Controller (Santa Clara, CA, USA), at 25 °C, in 1 cm cuvettes, in the wavelength range 250–700 nm. Stock solutions were prepared in DMSO and diluted with CH_2_Cl_2_, with the final concentration of DMSO below 1%, in concentration ranges of 10^−4^–10^−6^ M for the determination of the molar extinction coefficient (ε). Fluorescence measurements were performed in a Varian Cary Eclipse fluorimeter (Crawley, UK), equipped with a constant temperature cell holder, at 25 °C, in 1 cm cuvettes. Spectra were recorded with excitation and emission slit widths of 5 nm and 550 V of voltage and by using the appropriate excitation wavelengths (*λ*_exc_). To minimize reabsorption effects, the absorbance’s sample values were kept below 0.1.

3-benzyloxy-1-(3′-carboxypropyl)-2-methyl-4-pyridinone (**L^1^**) was prepared as described in literature [31].

### 3.1. Synthesis of Rosamine ***1*** and Dihydrorosamine ***2***

Rosamine **1**: A solution of benzaldehyde (87 μL, 0.86 mmol, 1.0 equiv.), 3-aminophenol (0.1877 g, 1.72 mmol, 2.0 equiv.), and methanosulfonic acid (MeSO_3_H, 5 mL) was heated to 150 °C for ca. 16 h. After cooling to room temperature, the reaction mixture was precipitated in diethyl ether (100 mL) at 0 °C. The resulting residue was purified by flash chromatography using a mixture of CHCl_3_/MeOH (9:1) as eluent, to yield rosamine **1** as a yellow solid (124 mg, 50% of yield). ^1^H NMR (400.15 MHz, CD_3_OD) *δ* 6.84 (d, *J* 2.1 Hz, 2H, H-4, 5), 6.88 (dd, *J* 9.2 and 2.1 Hz, 2H, H-2, 7), 7.28 (d, *J* 9.2 Hz, 2H, H-1, 8), 7.44–7.46 (m, 2H, H*_ortho_*-Ph), 7.66–7.68 (m, 3H, H*_meta+para_*-Ph) ppm. ^13^C NMR (100.15 MHz, CD_3_OD) *δ* 97.2 (C-4 and C-5), 113.2 (C-1a and C-8a), 116.5 (C-2 and C-7), 128.5 and 129.2 and 129.9 (C-Ph), 132.2 (C-1 and C-8), 158.5, 158.7, 159.9 (C-4a and C-5a) ppm. HRMS (ESI) *m/z*: [M]^+^ calcd. for C_19_H_15_N_2_O^+^ 287.1179, found 287.1182.

Dihydrorosamine **2**: Rosamine **1** (36.0 mg, 0.125 mmol) was hydrogenated using H_2_ (4.5 bar), a catalytic amount of 10% Pd/C (w/w and HCl (37%, 10 drops) in MeOH (4 mL) for ca. 16 h. The resulting mixture was filtered to remove the catalyst, washed several times with methanol and chloroform, then the solvent was evaporated under reduced pressure. Dihydrorosamine was obtained as a pale-yellow solid (36.1 mg, quantitative yield). ^1^H NMR (400.15 MHz, CD_3_OD) *δ* 5.48 (s, 1H, H-9), 7.12 (dd, *J* 8.0 and 2.2 Hz, 2H, H-2, 7), 7.20–7.25 (m, 3H, H-Ar), 7.28–7.35 (m, 6H, H-Ar) ppm. ^13^C NMR (100.15 MHz, CD_3_OD) *δ* 43.0 (C-9), 111.2, 118.3, 125.4, 126.9, 127.8, 128.7, 130.3, 131.6, 145.6, 151.0 ppm. HRMS (ESI) *m/z*: [M+H]^+^ calcd. for C_19_H_17_N_2_O^+^ 289.1335, found 289.1341.

### 3.2. Synthesis of Conjugates ***3*** and ***4***

Condensation of **2** with **L^1^**: *N*-(3-Dimethylaminopropyl)-*N*′-ethylcarbodiimide hydrochloride (EDC, 62.8 mg, 0.328 mmol, 2.6 equiv.) was added to a solution of **L^1^** (93.3 mg, 0.325 mmol, 2.6 equiv.) in a 1:1 mixture of anhydrous Py/ DMF (0.6 mL) under N_2_ atmosphere. After stirring at room temperature for 2 h, dihydrorosamine **2** (36.1 mg, 0.125 mmol) was added and the reaction was maintained for 48 h, at room temperature, under N_2_ atmosphere. Afterwards, ethyl acetate was added, and the reaction mixture was maintained at −4 °C for two days. Then, the solvent was removed by decantation. The resulting residue was purified by preparative thin-layer chromatography using a mixture of CHCl_3_/MeOH (9:1) as eluent to yield **3** (1.1 mg, 2% of yield) and **4** (9.2 mg, 16% of yield), both isolated as pale-yellow solids.

Conjugate **3** ^1^H NMR (400.15 MHz, CD_3_OD) *δ* 2.27 (s, 3H, CH_3_), 2.77 (t, *J* 6.4 Hz, 2H, CH_2_CH_2_CONH), 4.34 (t, *J* 6.4 Hz, 2H, CH_2_CH_2_CONH), 5.03 (s, 2H, CH_2_C_6_H_5_), 5.20 (s, 1H, H-9), 5.77 (dd, *J* 9.2 and 2.8 Hz, 1H, H-xanthene), 6.39–6.42 (m, 1H, H-xanthene), 6.46 (d, *J* 7.6 Hz, 1H, H-5″), 6.98–7.18 (m, 5H, H-xanthene or H-Ar), 7.22–7.30 (m, 5H, H-xanthene or H-Ar), 7.37 (dd, *J* 7.6 and *J* 1.4 Hz, 2H, H-xanthene or H-Ar), 7.47 and 7.59 (2s broad, 2H, H-xanthene or H-Ar), 7.73 (d, *J* 7.6 Hz, 1H, H-6″) ppm. ^13^C NMR (100.15 MHz, CD_3_OD) *δ* 12.3 (CH_3_), 37.7 (CH_2_), 44.2 (C-9), 50.6 (CH_2_), 74.1 (CH_2_C_6_H_5_), 108.4, 108.5, 115.7, 115.9, 116.9, 121.3, 127.1, 127.5, 128.7, 128.8, 128.9, 129.2, 129.6, 130.6, 131.9, 137.9, 138.7, 139.0, 140.9, 144.5, 146.7, 147.6, 151.8, 165.6, 169.4 (CONH), 174.4 (C-4″) ppm. HRMS (ESI) *m/z:* [M+H]^+^ calcd. for C_35_H_32_N_3_O_4_^+^ 558.24, found 558.24.

Conjugate **4** ^1^H NMR (400.15 MHz, CD_3_OD) *δ* 2.27 (s, 6H, 2×CH_3_), 2.77 (t, *J* 6.7 Hz, 4H, 2×CH_2_CH_2_CONH), 4.35 (t, *J* 6.7 Hz, 4H, 2×CH_2_CH_2_CONH), 5.04 (s, 4H, 2×CH_2_C_6_H_5_), 5.19 (s, 1H, H-9), 6.45–6.48 (m, 3H, H-5″ and H-Ar), 6.97–7.13 (m, 4H), 7.28–7.41 (m, 14H), 7.47 (d, *J* 2.0 Hz, 1H), 7.54 (d, *J* 7.1 Hz, 1H), 7.73 (d, *J* 7.4 Hz, 2H, 2×H-6″) ppm. ^13^C NMR (150.92 MHz, CD_3_OD) *δ*: 11.4 (CH_3_), 36.8 (CH_2_), 43.3 (C-9), 49.7 (CH_2_), 72.9 and 73.2 (CH_2_C_6_H_5_), 107.5, 114.8, 116.97 and 116.02, 120.4, 127.8, 127.89 and 127.92, 127.97 and 127.99, 128.3, 128.7, 128.8, 129.7, 137.0, 137.3, 140.0, 143.6, 145.8, 150.9, 161.5, 161.7, 161.9, 168.5 (CONH), 173.6 (C-4″) ppm. HRMS (ESI) *m/z*: [M+H]^+^ calcd. for C_51_H_47_N_4_O_7_^+^ 827.3439, found 827.3463.

### 3.3. Synthesis of Conjugates ***5*** and ***6***

Condensation of **Rho110** and **L^1^**:

(a) Activation with EDC using 1.2 equiv. of **L^1^**. A solution of **L^1^** (36.5 mg, 0.127 mmol, 1.2 equiv.) and EDC (26.4 mg, 0.138 mmol, 1.3 equiv.) in a 1:1 mixture of anhydrous Py/DMF (2 mL) was stirred at room temperature for 30 min, under N_2_ atmosphere. Afterwards, **Rho110** (35.0 mg, 0.106 mmol) was added and the reaction was maintained for 24 h. Afterwards, ethyl acetate was added and the reaction mixture was maintained at −4 °C overnight. Then, the solvent was removed by decantation. The resulting residue was purified by preparative thin-layer chromatography using a mixture of CHCl_3_/MeOH (85:15) as eluent, to give a less polar fraction corresponding to **5** (19.7 mg, 31% of yield), followed by a fraction corresponding to the **Rho110** recovered (19.4 mg, 55% of yield) and traces of **6**.

(b) Activation with EDC using 1.7 equiv. of **L^1^**. A solution of **L^1^** (51.7 mg, 0.180 mmol, 1.7 equiv.) and EDC (36.6 mg, 0.190 mmol, 1.8 equiv.) in a 1:1 mixture of anhydrous Py/DMF (2 mL) was stirred at room temperature for 30 min, under N_2_ atmosphere. Afterwards, **Rho110** (35.0 mg, 0.106 mmol) was added and the reaction was maintained for 24 h. The work-up was performed as described above to give **5** (25.4 mg, 40% of yield), followed by **Rho110** recovered and traces of **6**.

(c) Activation with EDC using 3.5 equiv. of **L^1^**. A solution of **L^1^** (93.7 mg, 0.326 mmol, 3.5 equiv.) and EDC (66.1 mg, 0.345 mmol, 3.7 equiv.) in a 1:1 mixture of anhydrous Py/DMF (0.6 mL) was stirred at room temperature under N_2_ atmosphere during 2 h. After that time, **Rho110** (31.7 mg, 0.086 mmol) was added and the reaction was maintained for 48 h. The work-up was performed as described above to give **5** (5.1 mg, 8% of yield) and **6** (25.5 mg, 34% of yield).

(d) Activation with HATU using 3.5 equiv. of **L^1^**. To a solution of **L^1^** (140 mg, 0.488 mmol, 3.5 equiv.) in DMF (2 mL), HATU (186 mg, 0.489 mmol, 3.5 equiv.) was added under N_2_ atmosphere. After stirring at room temperature for 5 min, DIPEA (230 µL, 1.32 mmol, 9.5 equiv.) was added, followed by a solution in DMF (1 mL) of **Rho110** (51 mg, 0.140 mmol, 1.0 equiv.). The reaction was stirred for 25 h. Then, ethyl acetate was added to precipitate the reaction crude, which was purified through preparative thin-layer chromatography using a mixture of MeOH/CHCl_3_ (2:8), allowing the isolation of **5** (17.5 mg, 21% of yield).

(e) Activation with thionyl chloride using 3.0 equiv. of **L^1^**. To a solution of **L^1^** (70 mg, 0.245 mmol, 3.0 equiv.) in DMF (3 mL), thionyl chloride (30 µL, 0.409 mmol, 5.0 equiv.) was added under N_2_ atmosphere. After stirring at room temperature for 1 h, DIPEA (43 µL, 0.245 mmol, 3.0 equiv.) was added, followed by **Rho110** (30 mg, 0.082 mmol, 1.0 equiv.). The reaction was stirred for 18 h. Then, diethyl ether was added to precipitate the reaction crude, which was purified through preparative thin-layer chromatography using a mixture of NH_3_/MeOH/ CHCl_3_ (1:19:80). The less polar fluorescent fraction was recovered corresponding to **5** (15.0 mg, 24% of yield).

Conjugate **5** ^1^H NMR (400.15 MHz, CD_3_OD) *δ* 2.29 (s, 3H, CH_3_), 2.82 (t, *J* 6.7 Hz, 2H, CH_2_CH_2_CONH), 4.38 (t, *J* 6.7 Hz, 2H, CH_2_CH_2_CONH), 5.056 and 5.060 (2s, 2H, CH_2_C_6_H_5_), 6.43 (dd, *J* 8.8 and 2.0 Hz, 1H, H-xanthene), 6.46–6.49 (m, 2H, H-xanthene and H-5″), 6.59 (d, *J* 2.0 Hz, 1H, H-xanthene), 6.69 (d, *J* 8.4 Hz, 1H, H-xanthene), 7.06 (dd, *J* 8.8 and 2.0 Hz, 1H, H-xanthene), 7.19 (d, *J* 7.2 Hz, 1H, H-xanthene), 7.31–7.34 and 7.38–7.41 (2m, 5H, H-Ar), 7.71–7.77 (m, 4H, H-Ar and H-6″), 8.00–8.03 (m, 1H, H-3′) ppm. ^13^C NMR (100.15 MHz, CD_3_OD) *δ* 11.4 (CH_3_), 36.8, 49.6, 73.2, 86.5, 100.2, 106.8, 107.1, 111.7, 114.7, 114.8, 116.0, 124.0, 124.5, 127.1, 127.9, 128.0, 128.1, 128.4, 128.8, 129.7, 135.0, 137.1, 140.0, 140.4, 143.6, 145.8, 151.3, 152.0, 152.5, 152.8, 168.8 (CONH), 170.2 (2′-COO), 173.6 (C-4″) ppm. HRMS (ESI) *m/z*: [M+H]^+^ calcd. for C_36_H_30_N_3_O_6_^+^ 600.2129, found 600.2168.

Conjugate **6** ^1^H NMR (400.15 MHz, CD_3_OD) *δ* 2.22 (s, 6H, 2×CH_3_), 2.52 (t, *J* 7.1 Hz, 4H, 2×CH_2_CH_2_CONH), 4.21 (t, *J* 7.1 Hz, 4H, 2×CH_2_CH_2_CONH), 5.06 (s, 4H, 2×CH_2_C_6_H_5_), 6.45 (d, *J* 7.2 Hz, 2H, 2×H-5″), 6.80 (dd, *J* 9.0 and 2.0 Hz, 2H, H-2, 7), 6.83 (d, *J* 2.0 Hz, 2H, H-4, 5), 7.03 (d, *J* 9.0 Hz, 2H, H-1, 8), 7.31–7.43 (m, 11H, 2×CH_2_C_6_H_5_ and H-6′), 7.76 (d, *J* 7.2 Hz, 2H, 2×H-6″), 7.80 and 7.86 (2dt, *J* 7.7 and 1.4 Hz, 2H, H-4′, 5′), 8.31 (dd, *J* 7.6 and 1.2 Hz, 1H, H-3′) ppm. ^13^C NMR (100.15 MHz, CD_3_OD) *δ* 11.4 (CH_3_), 37.5, 51.0, 51.5, 73.1, 97.0, 113.5, 115.9, 116.5, 127.9, 128.0, 128.8, 130.0, 130.1, 130.2, 130.8, 131.4, 132.7, 133.8, 137.1, 140.0, 143.7, 145.7, 158.3, 159.9, 160.1, 165.6 (2′-COO), 173.4 (C-4″), 175.7 (CONH) ppm. HRMS (ESI) *m/z*: [M+H]^+^ calcd. for C_52_H_45_N_4_O_9_^+^ 869.3181, found 869.3197.

### 3.4. Deprotection of Benzyl Ether of ***5***

(a) with boron trichloride: Boron trichloride (BCl_3_, 1.0 M in CH_2_Cl_2_, 0.2 mL) was added to a solution of **5** (23.8 mg, 3.97 × 10^−5^ mol) in anhydrous CH_2_Cl_2_ (3 mL) under N_2_ atmosphere at 0 °C. The reaction proceeded for 5 h. After washing with methanol and acetone and concentration by rotary evaporation under reduced pressure, the resulting residue was crystallized in a mixture of CHCl_3_ and EtOH (9:1) to yield **Rho110-monoHPO** in dihydroxypyridinium form (15.2 mg, 75% of yield), as an orange solid.

(b) via hydrogenolysis:

A solution of conjugate **5** (56.7 mg, 0.073 mmol) in methanol (5 mL) and HCl (0.01 mL) was placed into a hydrogenation vessel. The air was removed with N_2_, a catalytic amount of 10% Pd/C (*w/w*) was added and the mixture was stirred at room temperature, with H_2_ at 5 bar for 3 h. The reaction mixture was filtered and the solvent evaporated in vacuum to give the crude product. The resulting residue was crystallized in MeOH/CHCl_3_ to give the deprotected compound in the reduced form (H insertion in position 9 of the rhodamine). Keeping this derivative in solution at room temperature for 24 h, we found that it can be totally converted into the oxidized form (**Rho110-monoHPO)**, without any further treatment, affording 15.5 mg of **Rho110-monoHPO** (35% of yield) as an orange powder.

**Rho110-monoHPO** ^1^H NMR (400.15 MHz, CD_3_OD+DCl few drops) *δ* 2.75 (s, 3H, CH_3_), 3.24 (t, *J* 6.4 Hz, 2H, CH_2_CH_2_CONH), 4.82 (t, *J* 6.4 Hz, 2H, CH_2_CH_2_CONH), 7.05 (d, *J* 2.0 Hz, 1H), 7.12 (dd, *J* 9.2 and 2.0 Hz, 1H), 7.21 (d, *J* 7.2 Hz, 1H, H-5″), 7.23–7.27 (m, 2H), 7.47 (d, *J* 7.6 Hz, 1H), 7.56 (dd, *J* 9.2 and *J* 2.0 Hz, 1H), 7.86 (dt, *J* 7.7 and *J* 1.4 Hz, 1H), 7.92 (dt, *J* 7.5 and *J* 1.4 Hz, 1H), 8.31 (d, *J* 7.2 Hz, 1H, H-6″), 8.37 (d, *J* 7.2 Hz, 1H), 8.43 (d, *J* 1.6 Hz, 1H) ppm. ^13^C NMR (100.15 MHz, CD_3_OD) *δ* 11.5 (CH_3_), 36.1 (CH_2_), 51.6 (CH_2_), 97.4, 105.8, 110.3, 116.4, 116.8, 117.3, 117.8, 119.4, 128.5, 129.6, 129.7, 130.1, 130.4, 130.5, 130.9, 132.6, 132.9, 134.7, 138.4, 141.6, 146.2, 155.0, 159.7, 162.0 (C-4″), 166.8 (CONH), 169.1 (2′-COO) ppm. HRMS (ESI) *m/z*: M^+•^ calcd. for C_29_H_24_N_3_O_6_^+^ 510.1660, found 510.1662.

## 4. Conclusions

The synthesis of mono- and di-functionalized pyridinone–rhodamine derivatives was successfully achieved through an amide coupling strategy of the carboxypropyl pyridinone unit (**L^1^**) with two fluorophores: dihydrorosamine (**2**) and rhodamine 110 (**Rho110**). The reaction using dihydrorosamine **2**, afforded the benzylated forms of mono- and di-functionalized derivatives in a single reduced form (H in the position 9 of the xanthene), while the same reaction with **Rho110** furnished mono- and di-functionalized derivatives predominantly in spirolactone form with higher efficacy.

The solution studies reveal that the introduction of the pyridinone unit at amino groups in the rhodamine derivatives **5** and **6** does not influence the absorption and emission spectra in the quinoid forms. By adding a small amount of HCl to the solutions of **5** and **6**, the spirocyclic ring is opened providing the quinoid form of the dyes, which exhibit absorption and emission wavelengths in the range of 485–489 nm and 508–521 nm, respectively.

A larger Stokes shift (Δ*λ* = 32 nm) was observed for **5**, meaning that this derivative is less susceptible to self-quenching via energy transfer processes. Its deprotection of benzyl ether group was successfully achieved with BCl_3_ (and also by hydrogenolysis) affording the hydrochloride salt of **Rho110-monoHPO** in good yield. The fluorescence intensity of **Rho110-monoHPO** was studied in the presence of several metal ions, revealing higher sensitivity towards Fe(III), making it an interesting fluorogenic dye for future applications.

## Figures and Tables

**Table 1:** Photophysical properties of **Rho110**, compounds **1–4** and functionalized derivatives **5** and **6** (*λ* of absorption and emission and molar extinction coefficient (*ε*) and Stokes shift) in CH_2_Cl_2_ at 25 °C.

Compound	*λ*_abs_/nm	*ε* × 10^3^/M^−1^⋅cm^−1^	*λ*_em_/nm	Stokes Shift/nm
**1**	511	3.3	526	15
**2**	507	1.4	530	23
**3**	277	46.3	-	-
**4**	270	40.2	-	-
**Rho110**	498	89.0	515	17
**5** (spirolactone)	270	2.1	-	-
**6** (spirolactone)	262	79.8	-	-
**5** (quinoid)	489	0.8	521	32
**6** (quinoid)	485	18.7	508	23

## Data Availability

The data presented in this study are contained within the article and are also available in the Supplementary Material.

## Supplementary Materials

The following are available online: ^1^H NMR, ^13^C NMR, *g*HSQC, *g*HMBC, MS, UV-Vis, and fluorescence spectra. Figure S1: ^1^H NMR spectrum (400.15 MHz, CD_3_OD) of **1**, Figure S2: ^13^C NMR spectrum (100.15 MHz, CD_3_OD) of **1**, Figure S3: HSQC (^1^H, ^13^C) spectrum of **1**, Figure S4: HMBC (^1^H, ^13^C) spectrum of **1**, Figure S5: ^1^H NMR spectrum (400.15 MHz, CD_3_OD) of **2**, Figure S6: ^13^C NMR spectrum (100.15 MHz, CD_3_OD) of **2**, Figure S7: HSQC (^1^H, ^13^C) spectrum of **2**, Figure S8: HMBC (^1^H, ^13^C) spectrum of **2**, Figure S9: ^1^H NMR spectrum (400.15 MHz, CD_3_OD) of **3**, Figure S10: ^13^C NMR spectrum (100.15 MHz, CD_3_OD) of **3**, Figure S11: HSQC (^1^H, ^13^C) spectrum of **3**, Figure S12: HMBC (^1^H, ^13^C) spectrum of **3**, Figure S13: ^1^H NMR spectrum (400.15 MHz, CD_3_OD) of **4**, Figure S14: ^13^C NMR spectrum (150.92 MHz, CD_3_OD) of **4**, Figure S15: ^1^H NMR spectrum (400.15 MHz, CD_3_OD) of **5**, Figure S16: ^13^C NMR spectrum (100.15 MHz, CD_3_OD) of **5**; * denote residual solvent (ethanol), Figure S17: HSQC (^1^H, ^13^C) spectrum of **5**, Figure S18: HMBC (^1^H, ^13^C) spectrum of **5**, Figure S19: ^1^H NMR spectrum (400.15 MHz, CD_3_OD) of **6**, Figure S20: ^13^C NMR spectrum (100.15 MHz, CD_3_OD) of **6**, Figure S21: HSQC (^1^H, ^13^C) spectrum of **6**, Figure S22: HMBC (^1^H, ^13^C) spectrum of **6**, Figure S23: ^1^H NMR spectrum (400.15 MHz, CD_3_OD+DCl) of **Rho110-monoHPO**, Figure S24: ^13^C NMR spectrum (100.15 MHz, CD_3_OD) of **Rho110-monoHPO**, Figure S25: Comparison of the ^1^H NMR spectra of **1** and **2**, Figure S26: Aromatic region of the ^1^H NMR spectrum of mono- and disubstituted derivatives **3** and **4**, in CD_3_OD, Figure S27: Aromatic region of the ^1^H NMR spectrum of mono- and disubstituted derivatives **5** and **6**, in CD_3_OD, Figure S28: HRMS (ESI) of **1**, Figure S29: HRMS (ESI) of **2**, Figure S30: HRMS (ESI) of **3**, Figure S31: HRMS (ESI) of **4**, Figure S32: HRMS (ESI) of **5**, Figure S33: HRMS (ESI) of **6**, Figure S34: HRMS (ESI) of **Rho110-monoHPO**, Figure S35: Absorption (normalized) and emission spectra of **Rho110**, **1**–**4** in CH_2_Cl_2_ at 25 °C, Figure S36: Absorption and emission spectra of **5** and **6** in CH_2_Cl_2_ before (spirolactone form, full lines) and after an aliquot addition of HCl (37%) (quinoid form, dashed lines) at 25 °C.

## Sample Availability

Samples of compounds **1–6** and **Rho110-monoHPO** are available from the authors.
