# Supplementary material for: Functionalization of Rhodamine Platforms with 3-Hydroxy-4-pyridinone Chelating Units and Its Fluorescence Behavior towards Fe(III)
Source: Molecules. 2022 Feb 26;27(5):1567. doi: 10.3390/molecules27051567 (PMC8911962; doi:10.3390/molecules27051567)
Supplement: Supplementary file 1 [file molecules-27-01567-s001.zip › molecules-1600947-supplementary.pdf]

Supplementary Materials

Functionalization of Rhodamine Platform with 3-Hydroxy-4-pyridinone Chelating Units and Its Fluorescence Behavior towards Fe(III)

Carla Queirós, Sílvia Vinhas, Jéssica Oliveira, Andreia Leite *, Ana M. G. Silva * and Maria Rangel

Conteúdo

S1. NMR spectra of rosamine **1** 2

S2. NMR spectra of dihydrorosamine **2** 4

S3. NMR spectra of conjugate **3** 6

S4. NMR spectra of conjugate **4** 8

S5. NMR spectra of conjugate **5** 9

S6. NMR spectra of conjugate **6** 11

S7. NMR spectra of **Rho110_monoHPO** 13

S8. Comparison of the ^{1}H NMR spectra of rosamine **1** and dihydrorosamine **2** 14

S9. Comparison of the ^{1}H NMR spectra of mono- and disubstituted derivatives **3** and **4** 15

S10. Comparison of the ^{1}H NMR spectra of mono- and disubstituted derivatives **5** and **6** 15

S11. MS spectra of rosamines and conjugates 16

S12. Comparative absorption spectra of **Rho110**, **1**, **2**, **3** and **4** 20

S13. Comparative absorption and emission spectra of spirolactone and quinoid forms of **5** and **6** 20

S1. NMR spectra of rosamine **1**

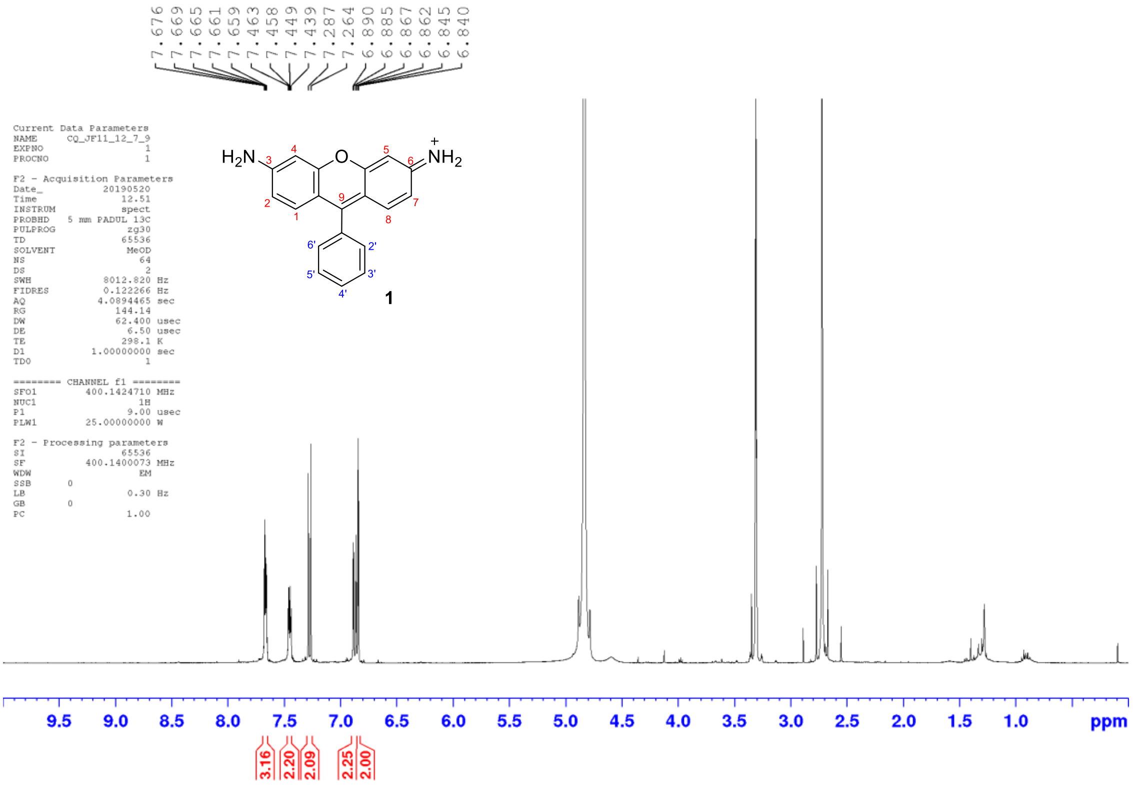

Figure S1. ^{1}H NMR spectrum (400.15 MHz, CD_3OD) of **1**.

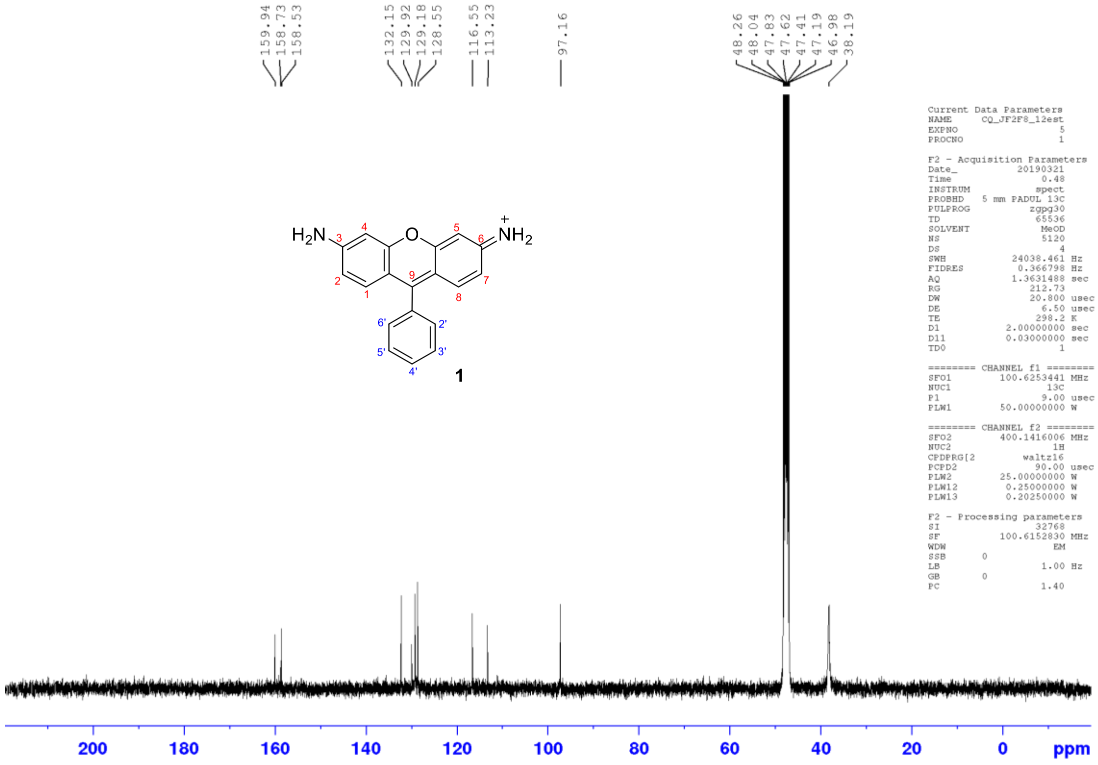

Figure S2. ^{13}C NMR spectrum (100.15 MHz, CD_3OD) of **1**.

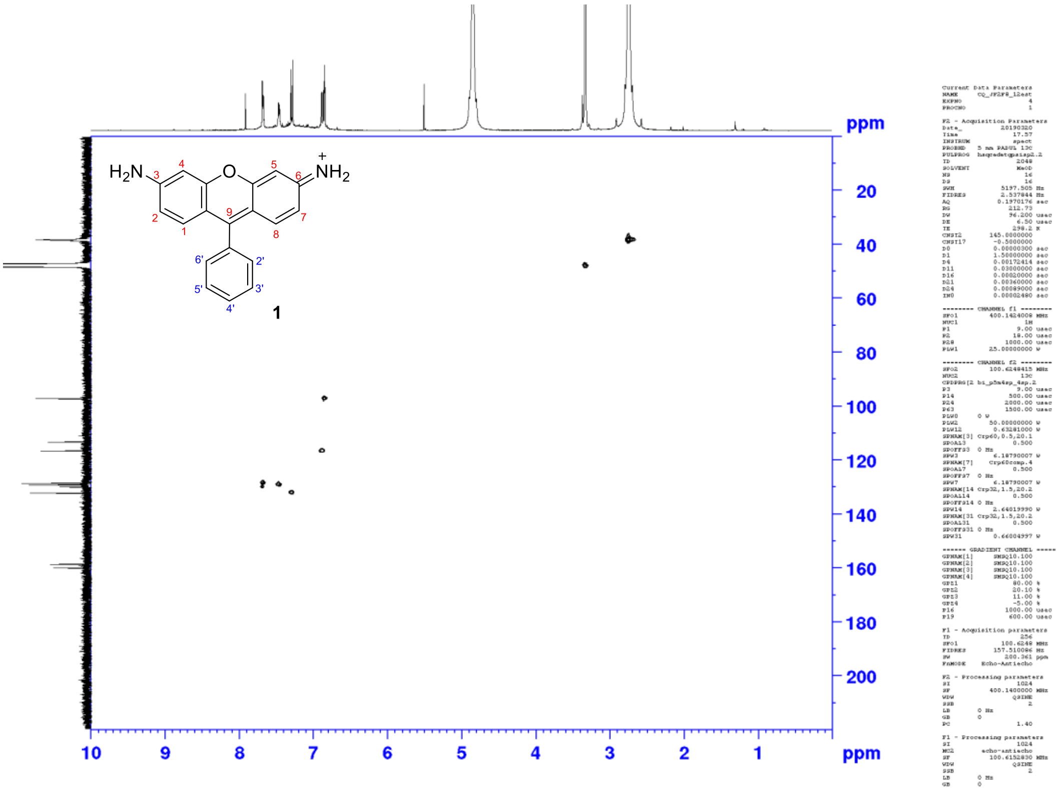

Figure S3. HSQC (^{1}H, ^{13}C) spectrum of **1**.

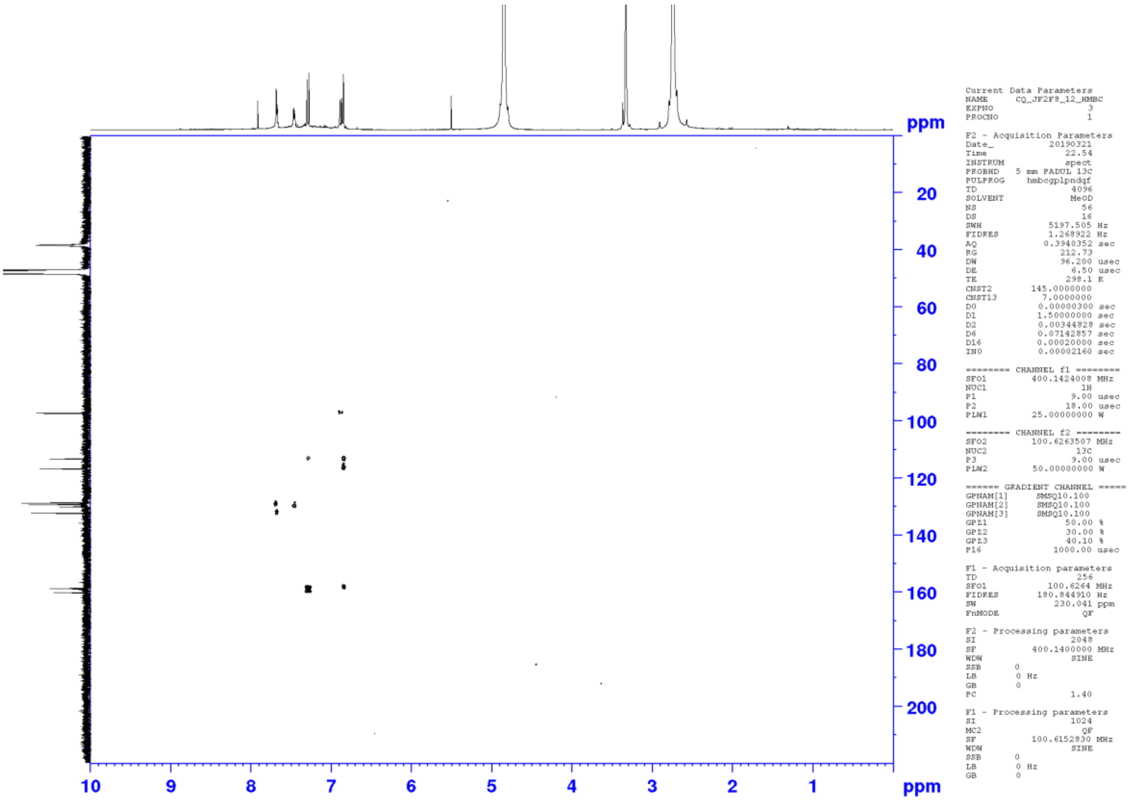

Figure S4. HMBC (^{1}H, ^{13}C) spectrum of **1**.

S2. NMR spectra of dihydrorosamine **2**

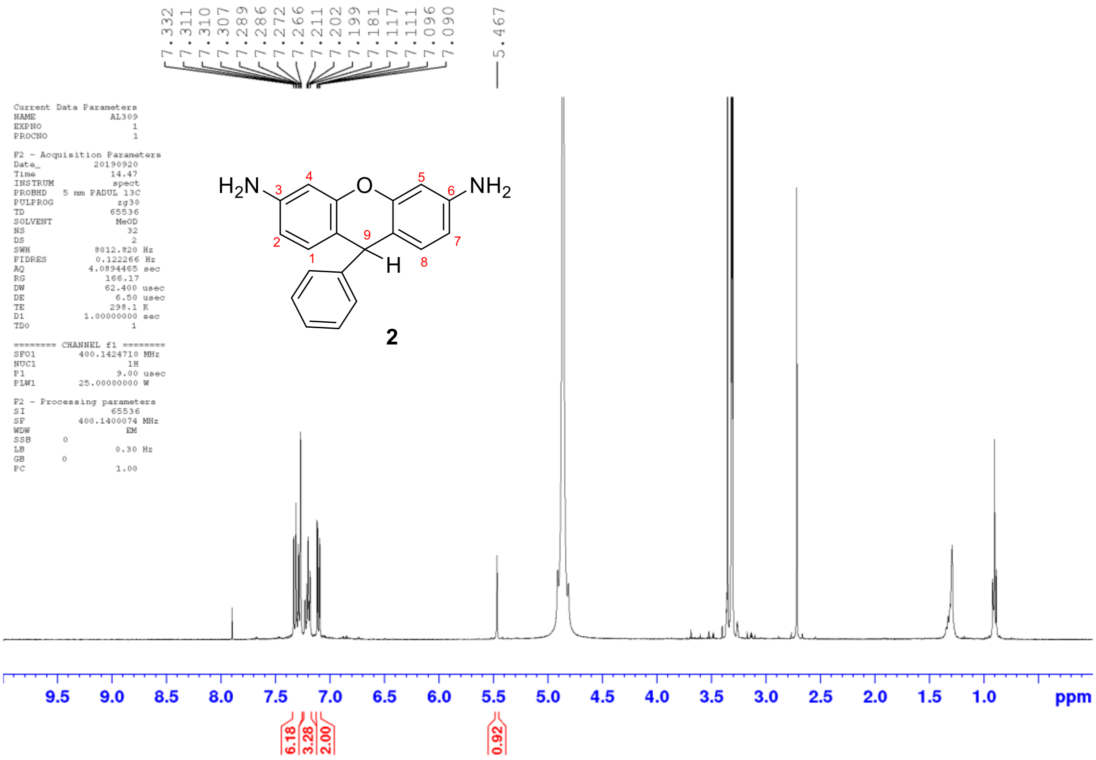

Figure S5. ^{1}H NMR spectrum (400.15 MHz, CD_3OD) of **2**.

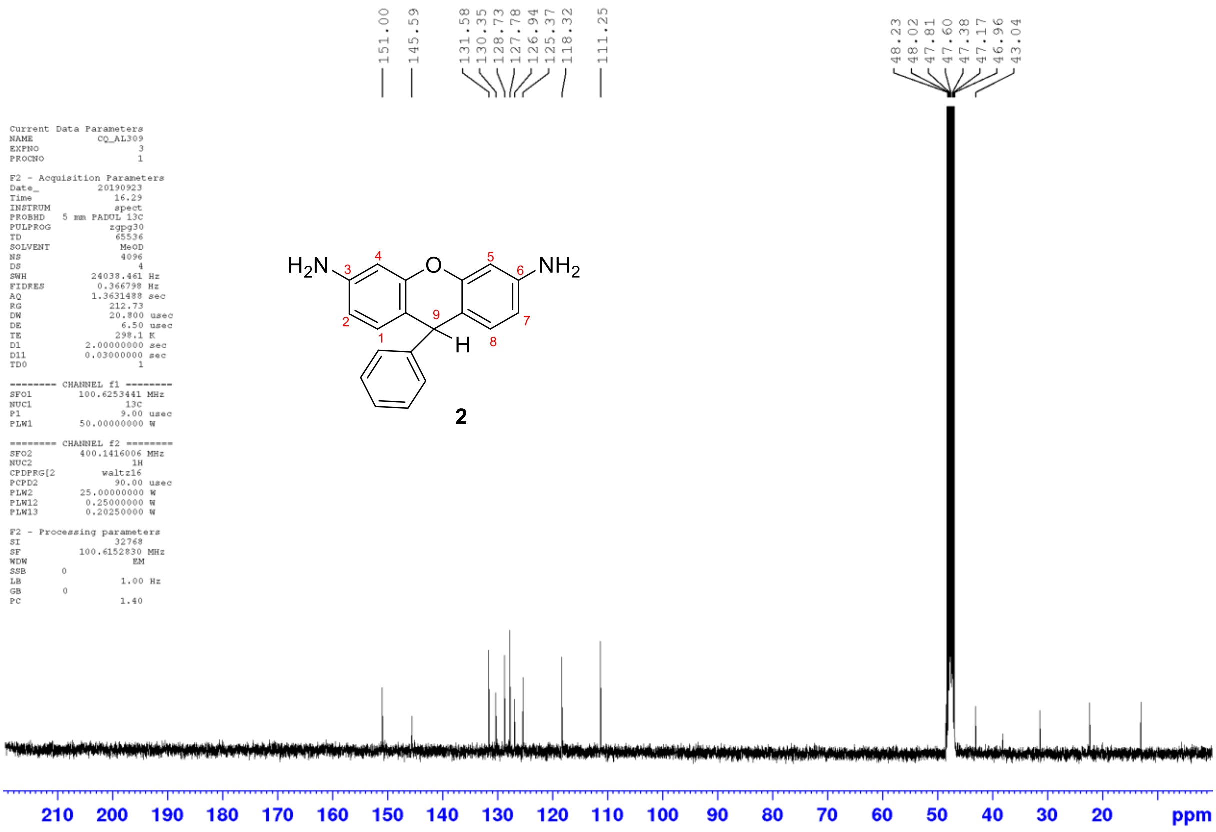

Figure S6. ^{13}C NMR spectrum (100.15 MHz, CD_3OD) of **2**.

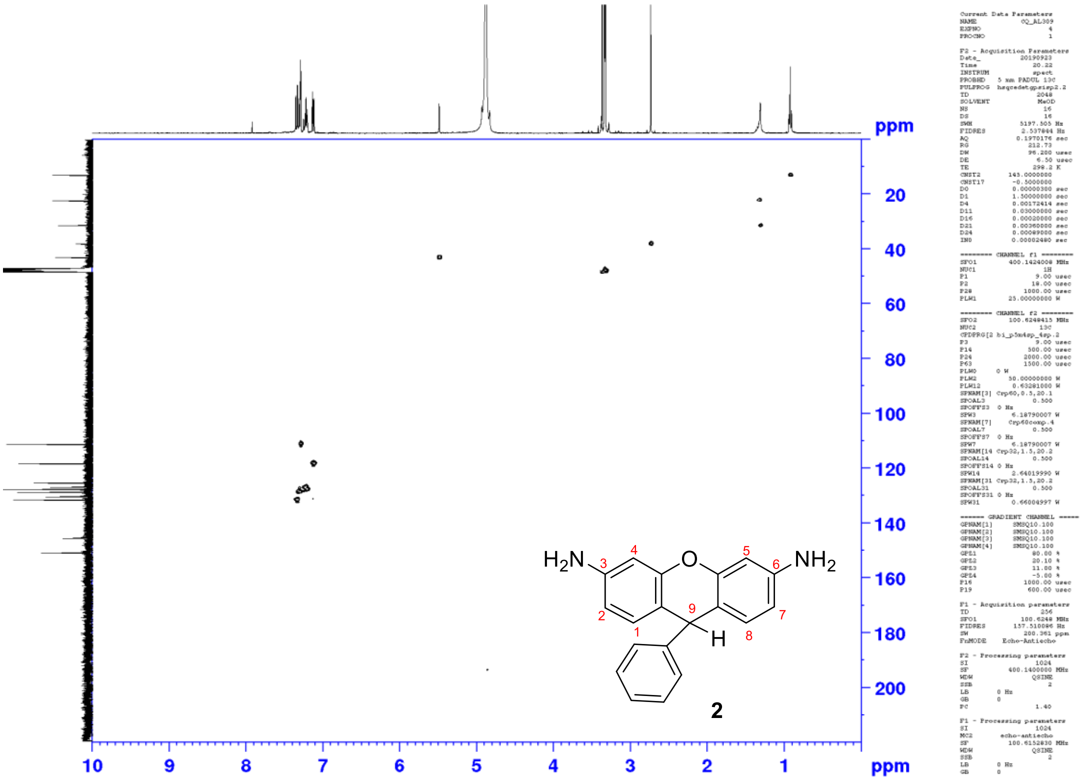

Figure S7. HSQC (^{1}H, ^{13}C) spectrum of **2**.

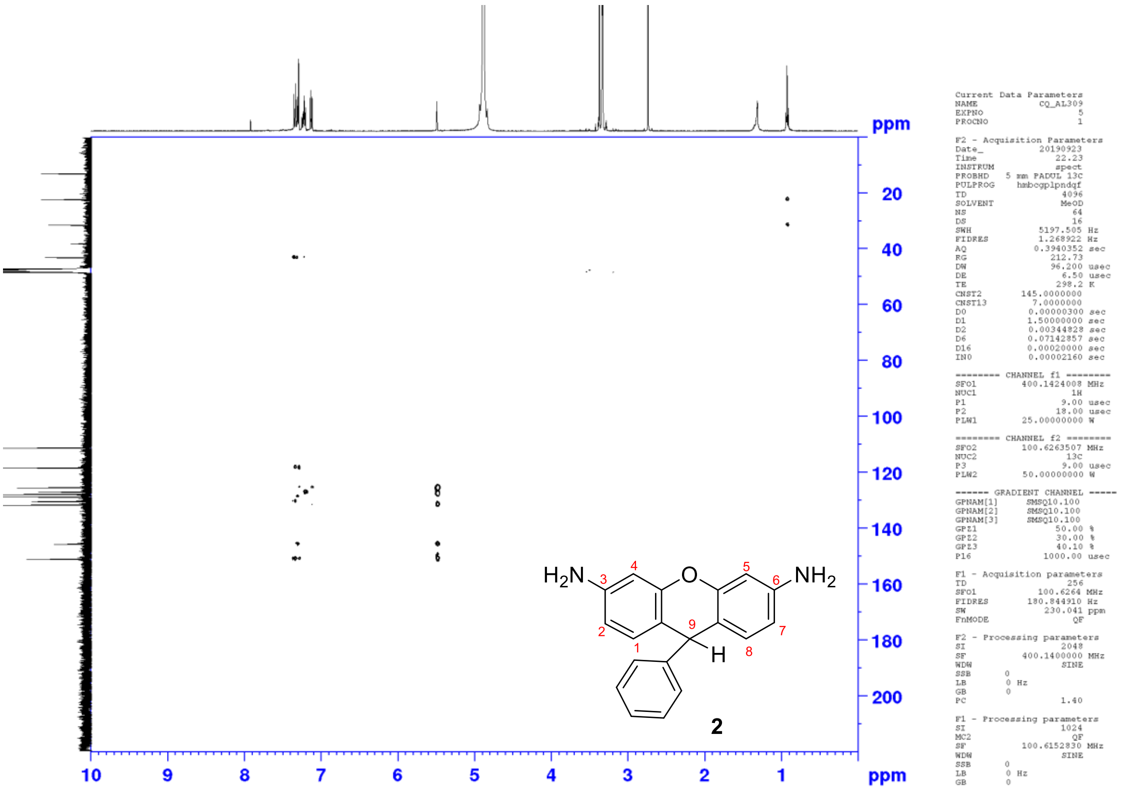

Figure S8. HMBC (^{1}H, ^{13}C) spectrum of **2**.

S3. NMR spectra of conjugate **3**

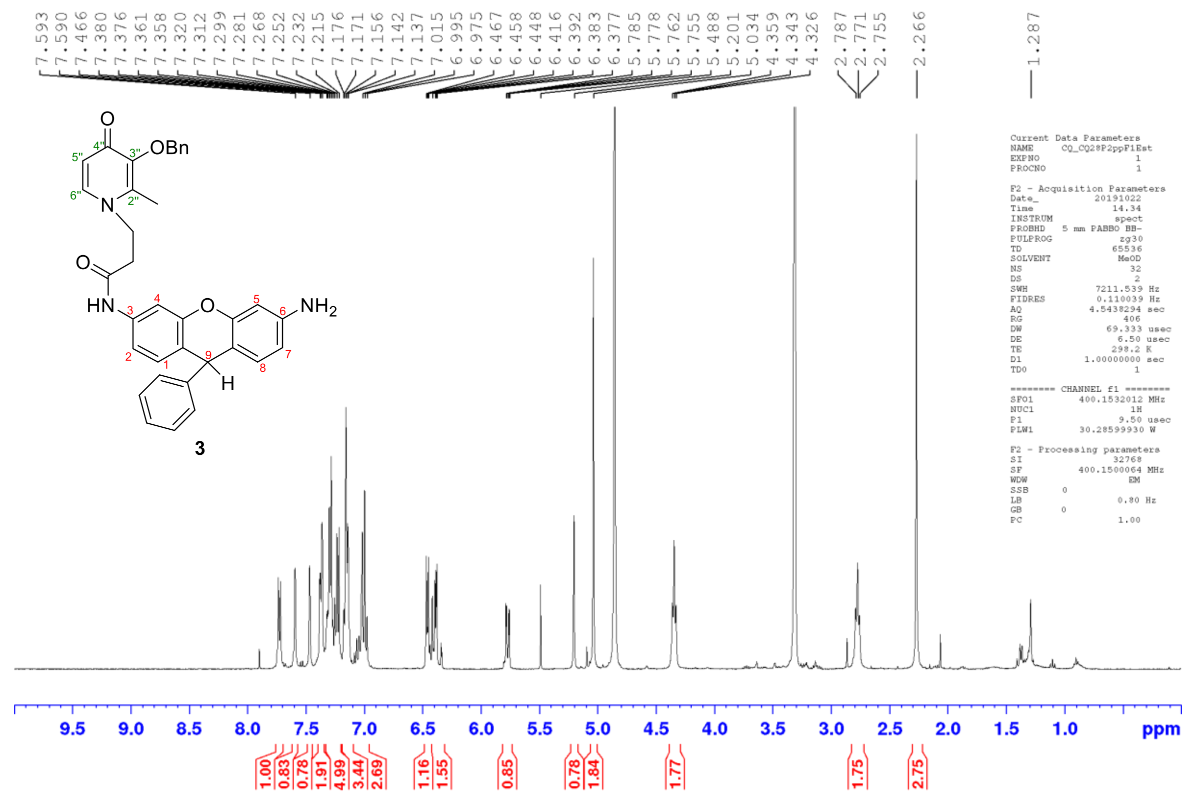

Figure S9. ^{1}H NMR spectrum (400.15 MHz, CD_3OD) of **3**.

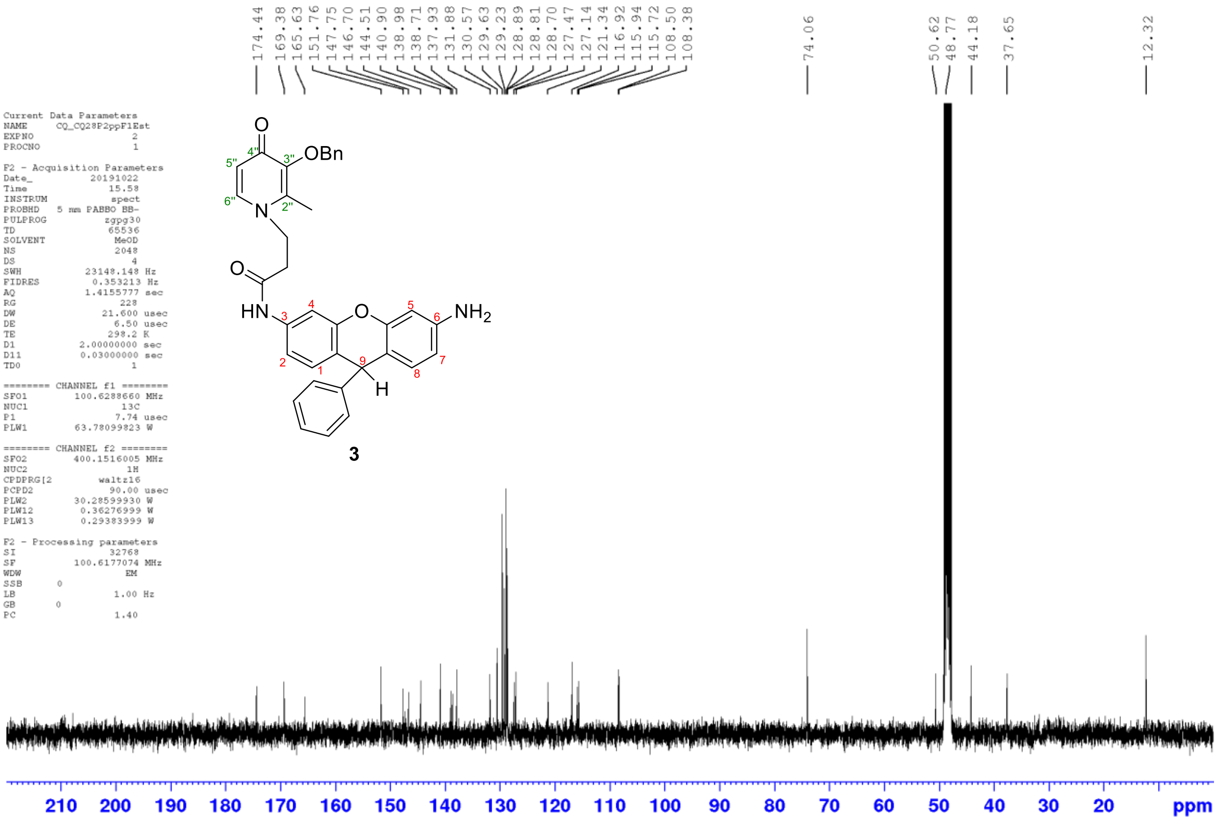

Figure S10. ^{13}C NMR spectrum (100.15 MHz, CD_3OD) of **3**.

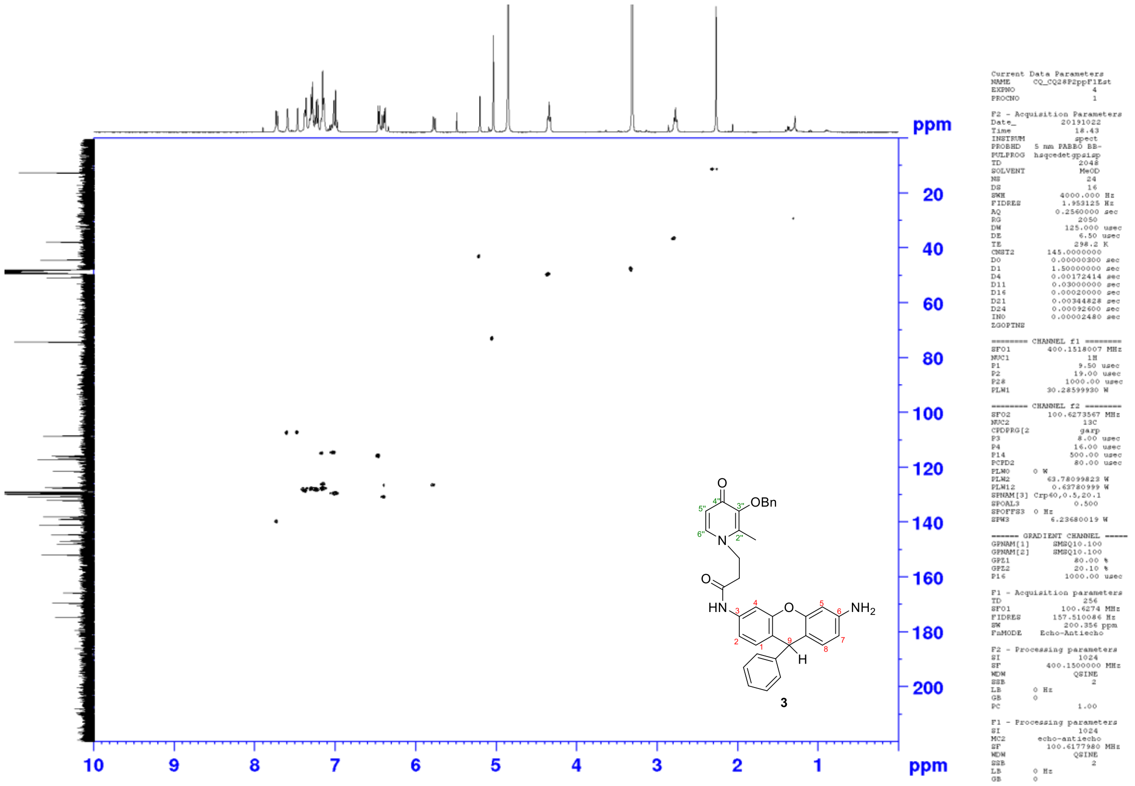

Figure S11. HSQC (^{1}H, ^{13}C) spectrum of **3**.

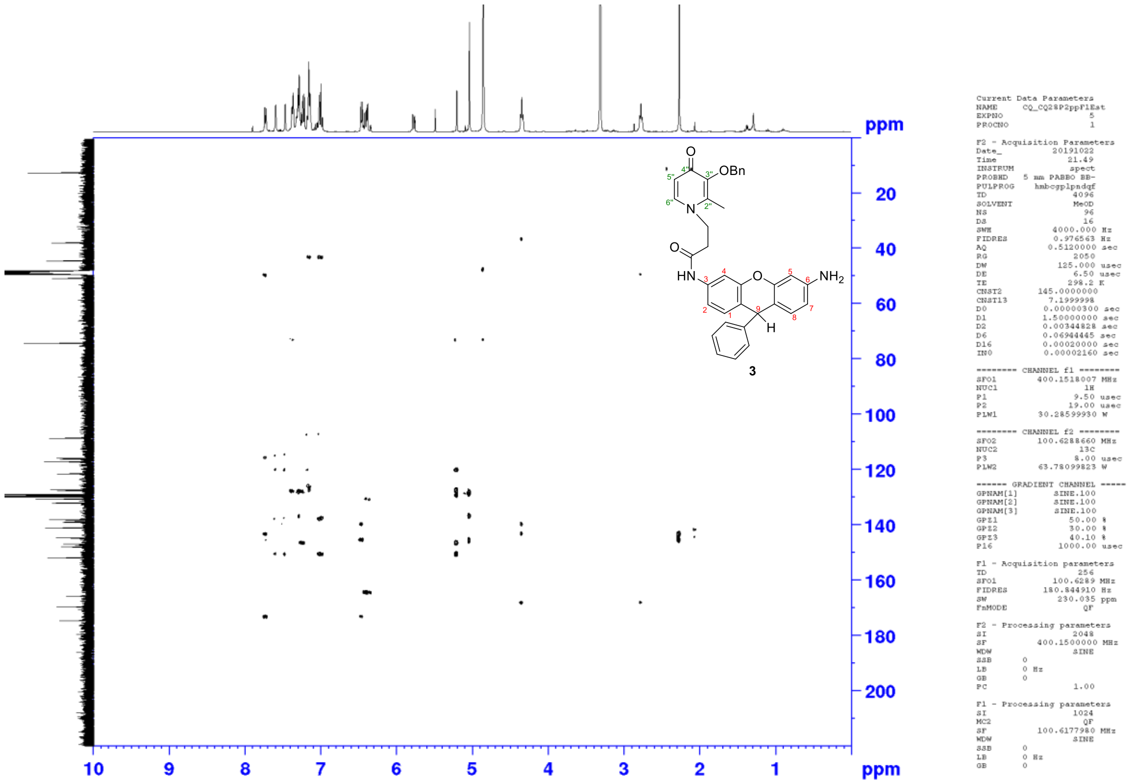

Figure S12. HMBC (^{1}H, ^{13}C) spectrum of **3**.

S4. NMR spectra of conjugate **4**

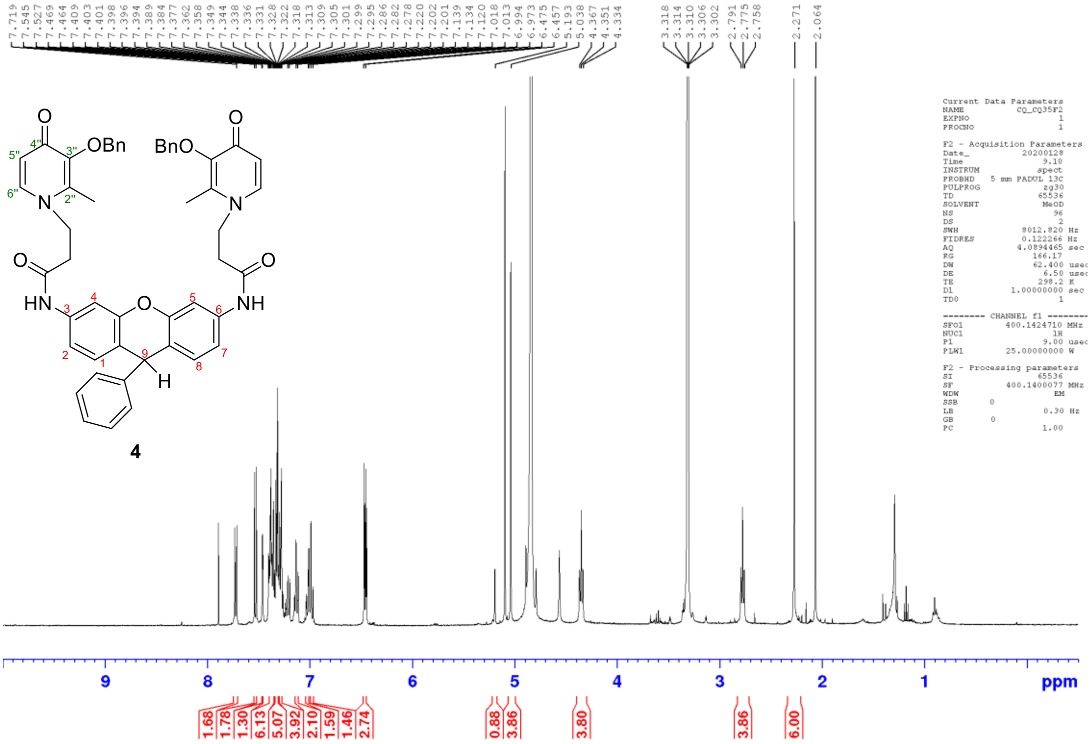

Figure S13. ^{1}H NMR spectrum (400.15 MHz, CD_3OD) of **4**.

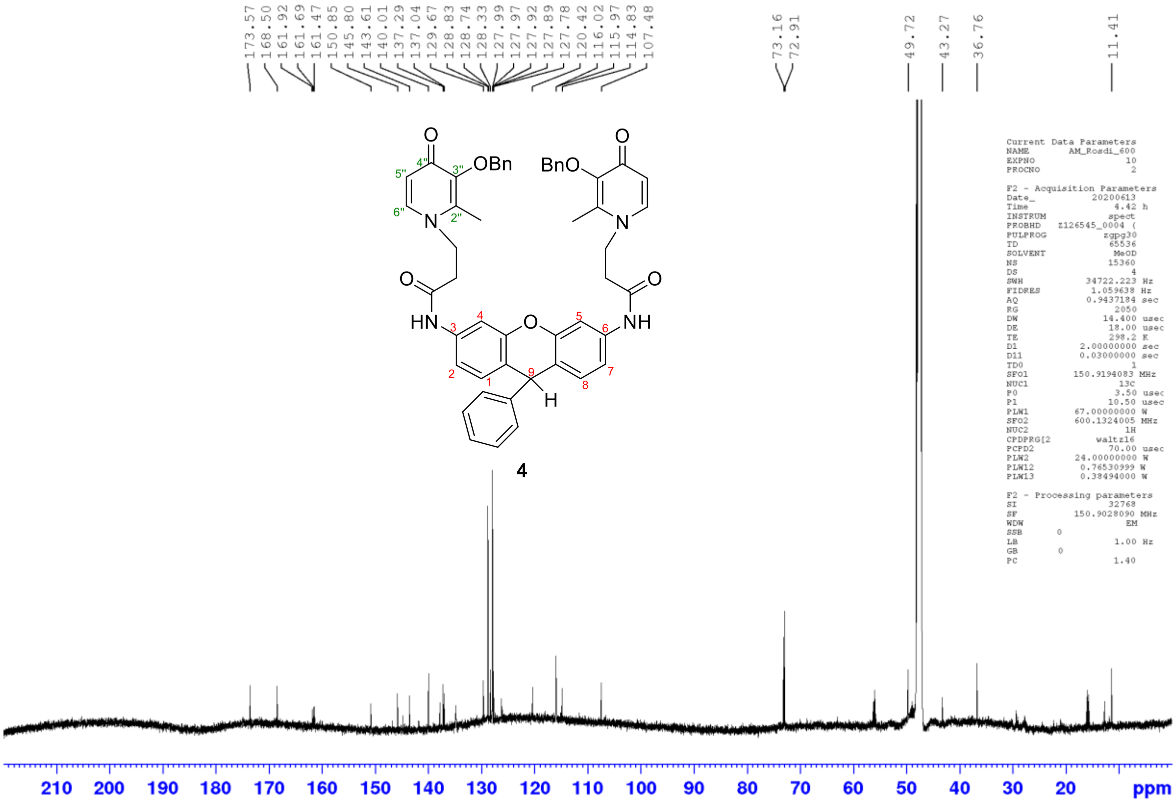

Figure S14. ^{13}C NMR spectrum (150.92 MHz, CD_3OD) of **4**.

S5. NMR spectra of conjugate **5**

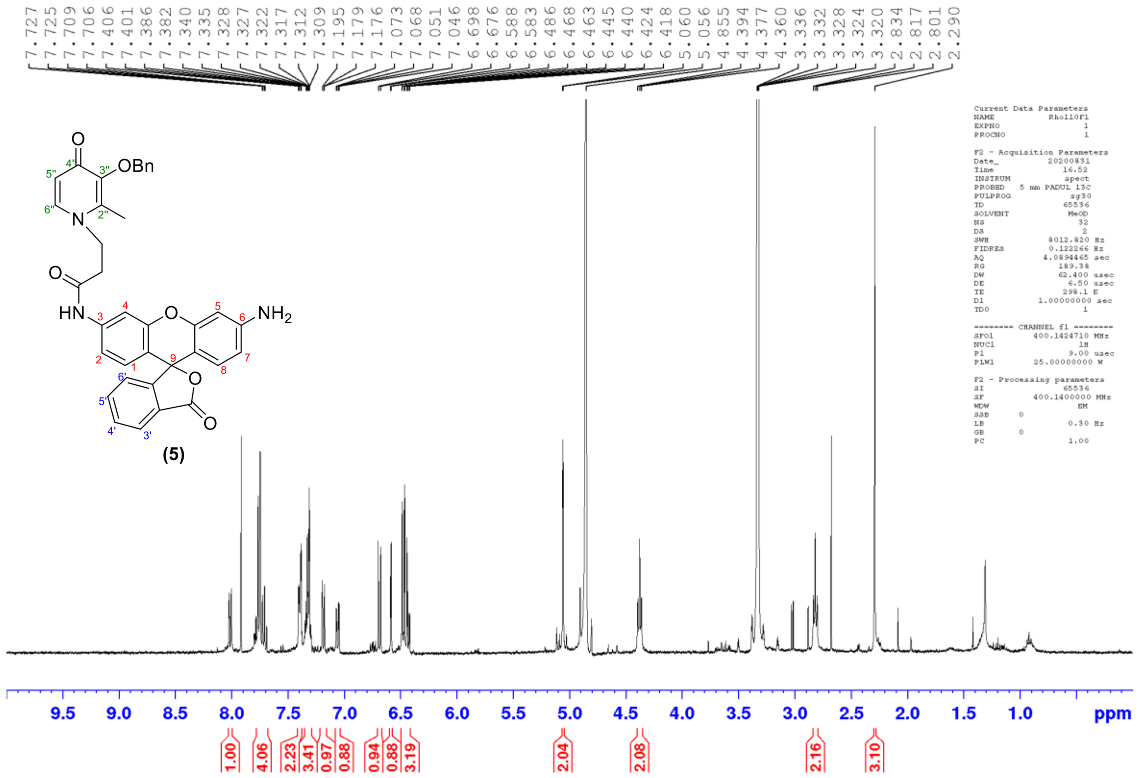

Figure S15. ^{1}H NMR spectrum (400.15 MHz, CD_3OD) of **5**.

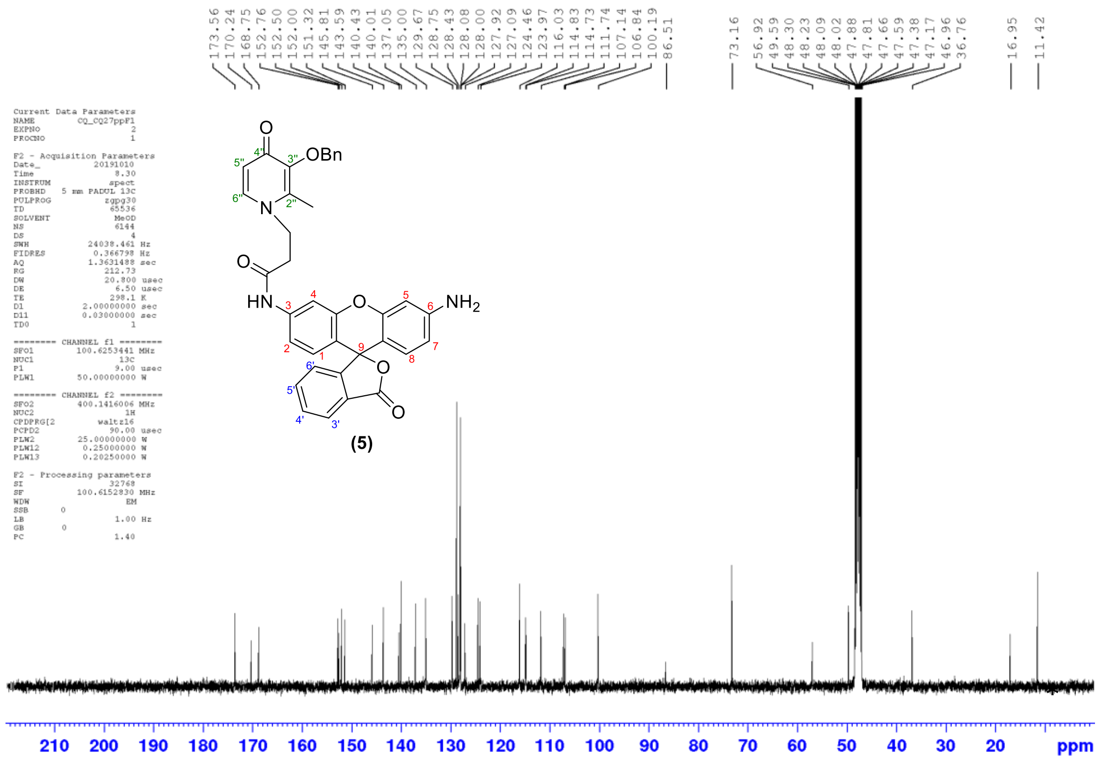

Figure S16. ^{13}C NMR spectrum (100.15 MHz, CD_3OD) of **5;** * denote residual solvent (ethanol).

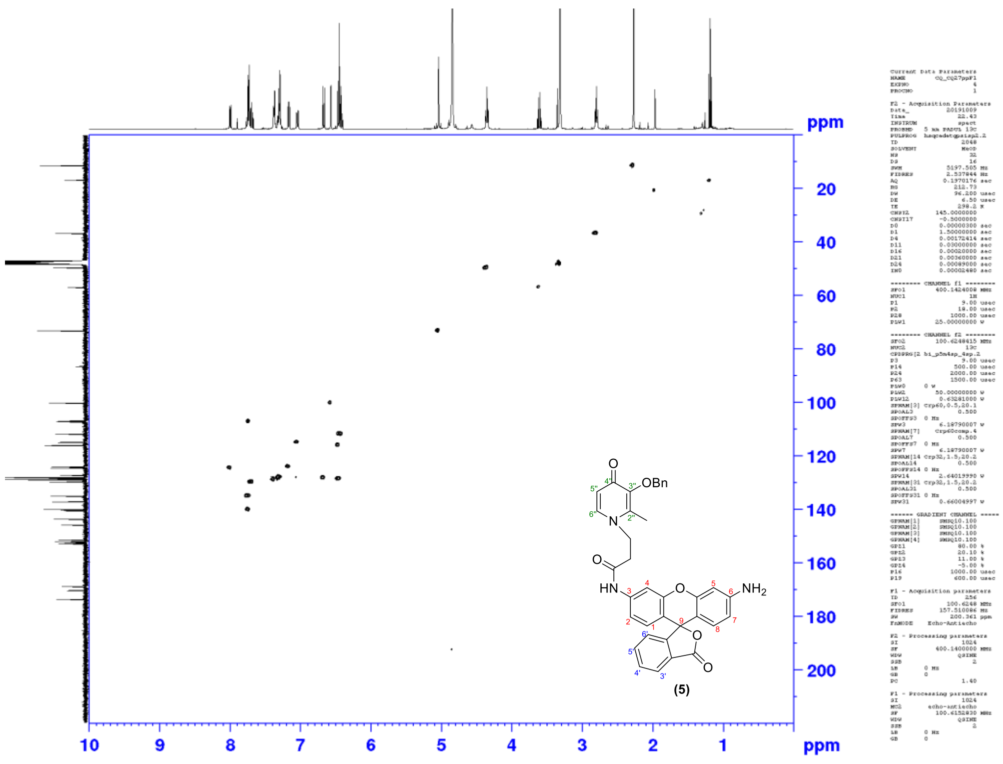

Figure S17. HSQC (^{1}H, ^{13}C) spectrum of **5**.

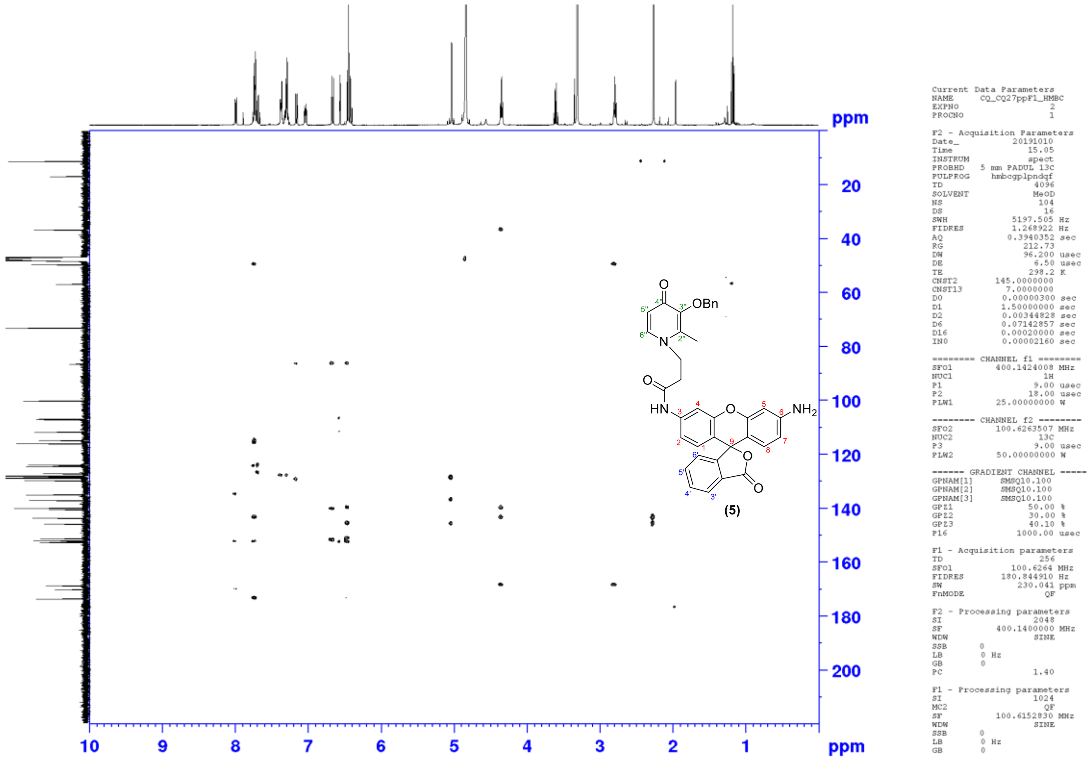

Figure S18. HMBC (^{1}H, ^{13}C) spectrum of **5**.

S6. NMR spectra of conjugate **6**

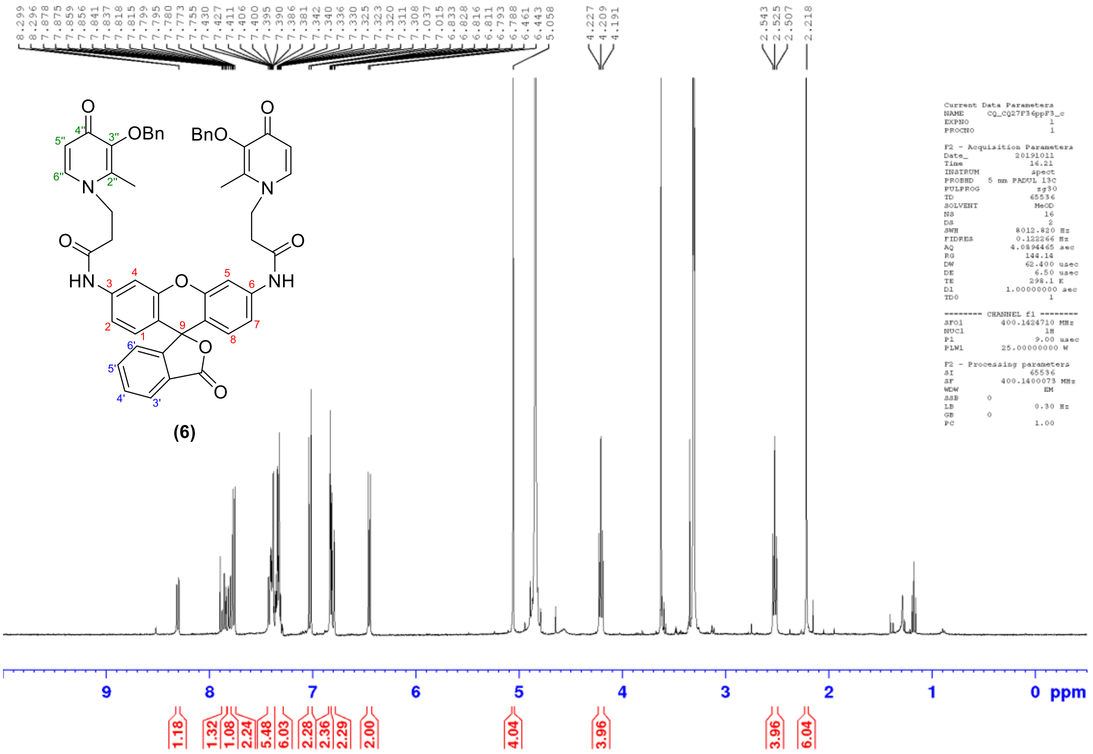

Figure S19. ^{1}H NMR spectrum (400.15 MHz, CD_3OD) of **6**.

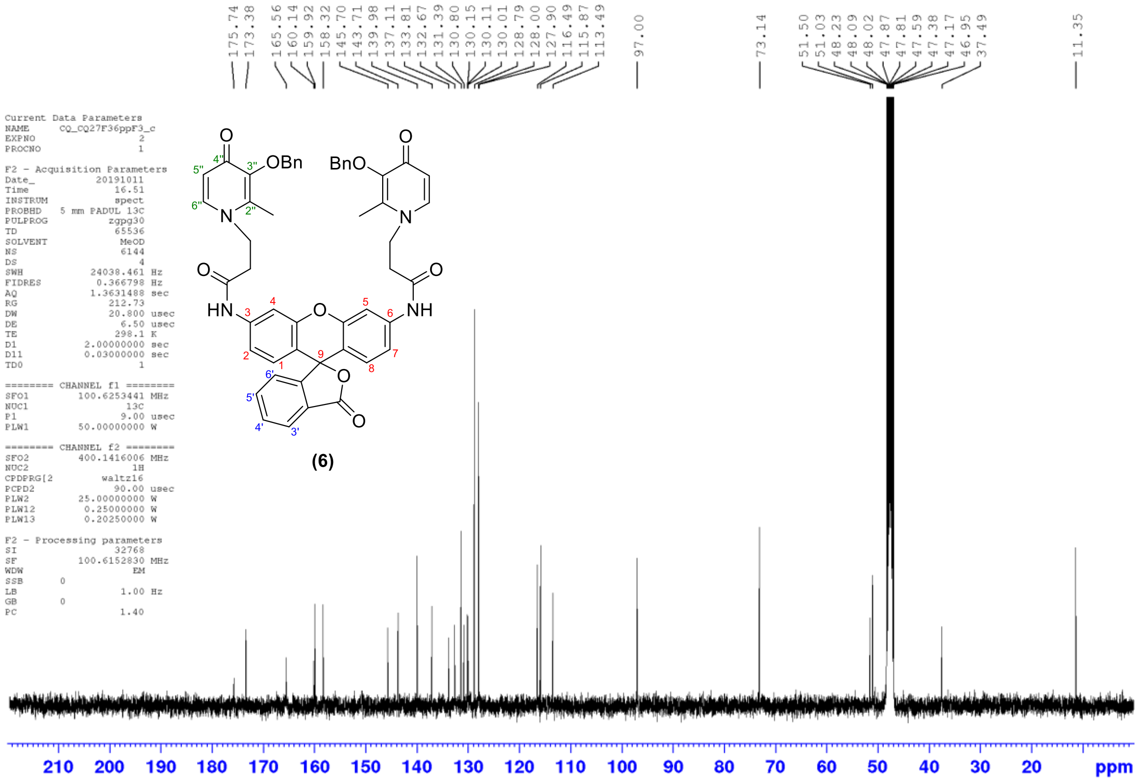

Figure S20. ^{13}C NMR spectrum (100.15 MHz, CD_3OD) of **6**.

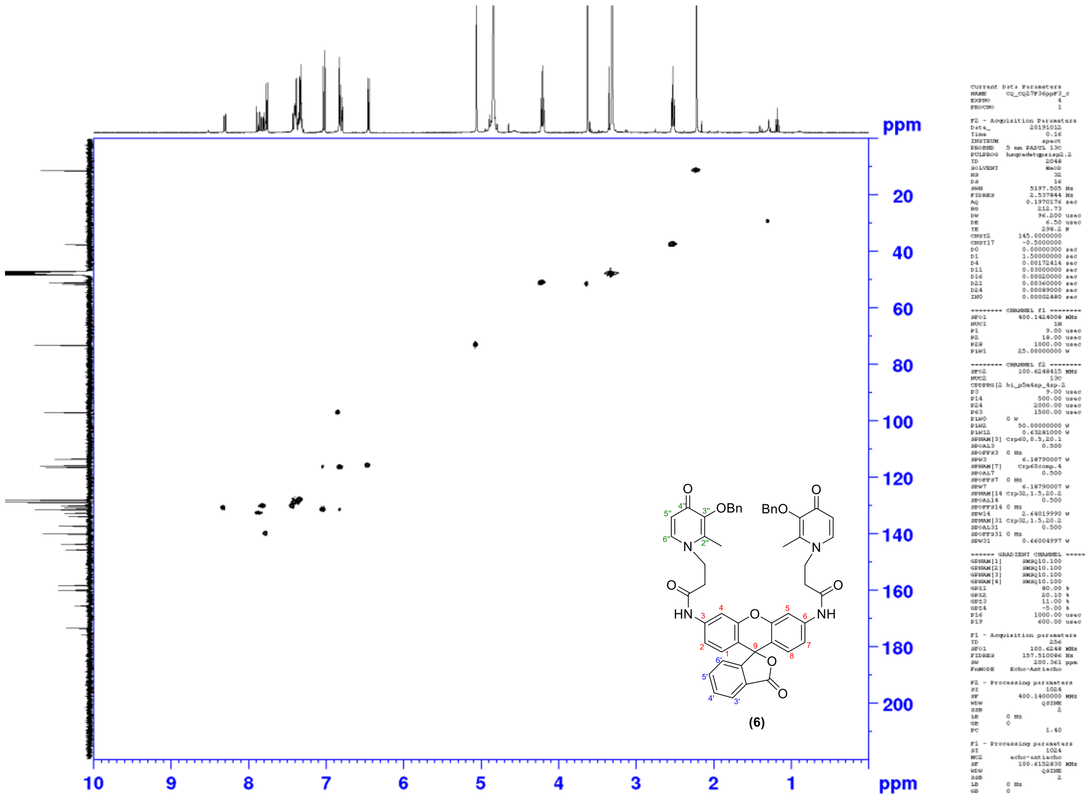

Figure S21. HSQC (^{1}H, ^{13}C) spectrum of **6**.

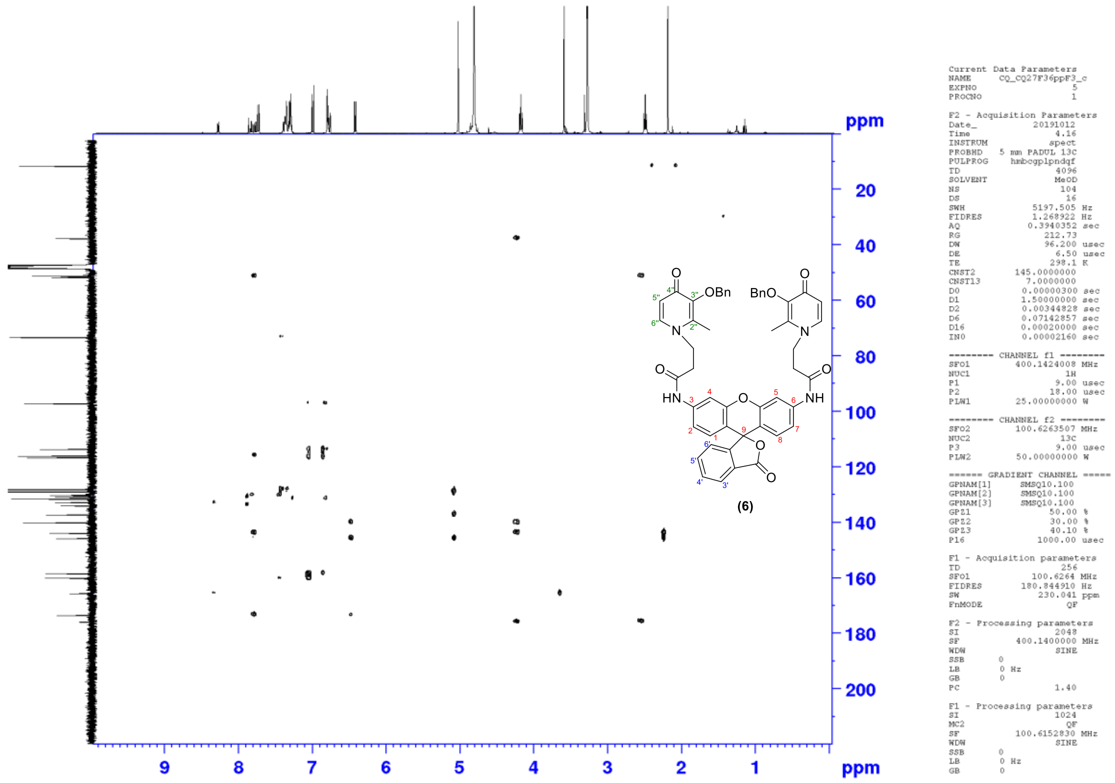

Figure S22. HMBC (^{1}H, ^{13}C) spectrum of **6**.

S7. NMR spectra of **Rho110_monoHPO**

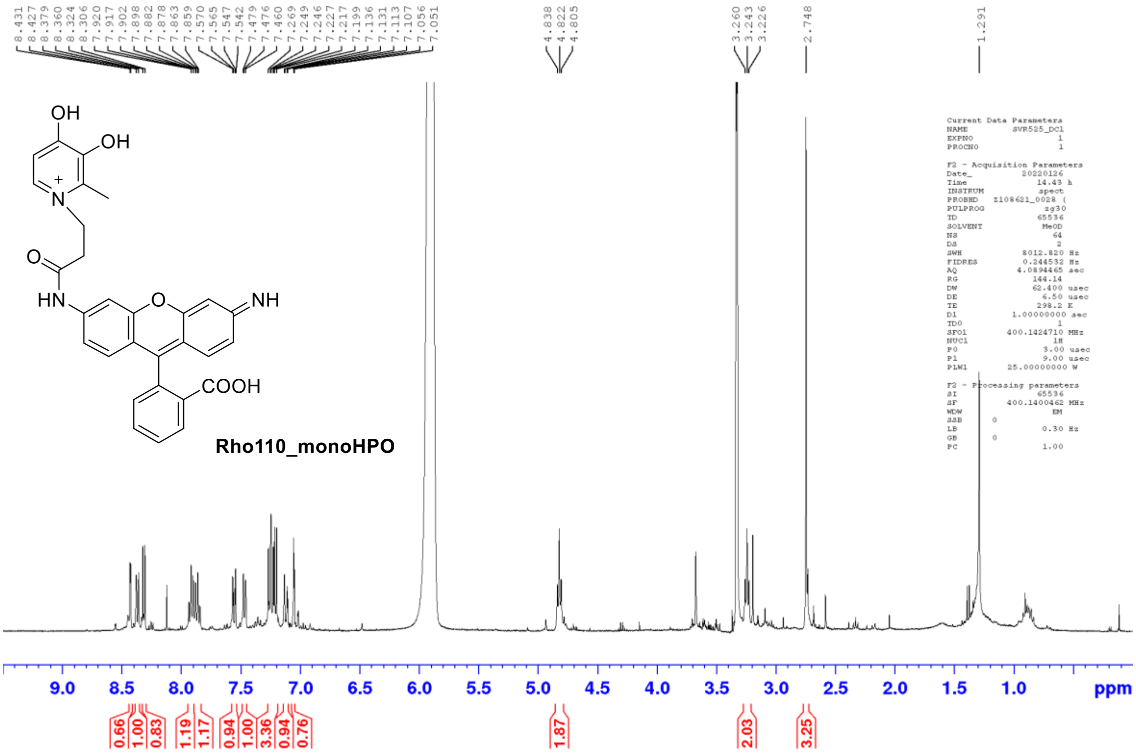

Figure S23. ^{1}H NMR spectrum (400.15 MHz, CD_3OD+DCl) of **Rho110-monoHPO**.

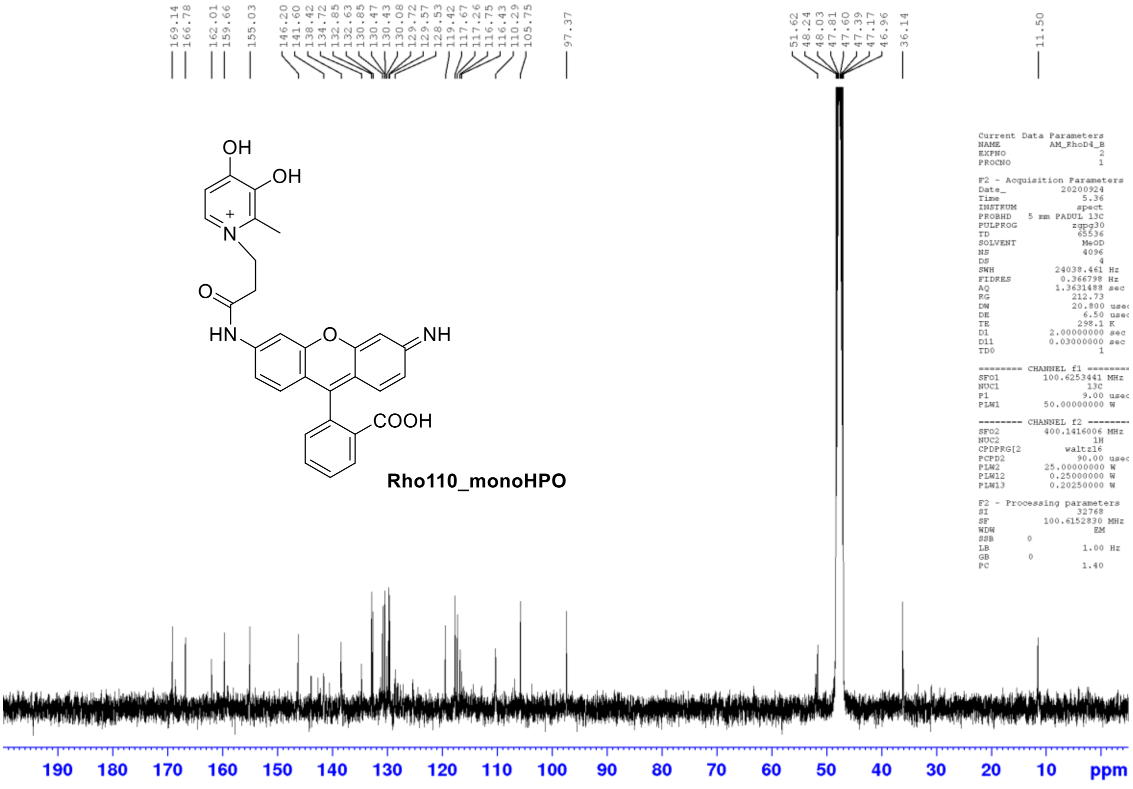

Figure S24. ^{13}C NMR spectrum (100.15 MHz, CD_3OD) of **Rho110-monoHPO**.

.

S8. Comparison of the ^{1}H NMR spectra of rosamine **1** and dihydrorosamine **2**

According to Figure S25, the ^{1}H NMR spectrum of rosamine **1** shows three signals at 6.84-7.28 ppm, that are typical of the xanthene scaffold and two signals at 7.44-7.68 ppm, attributed to the phenyl ring. After hydrogenation, the ^{1}H NMR spectrum of the resulting dihydrorosamine **2** shows an additional singlet at 5.48 ppm corresponding to the resonance of 9-H proton, together with slight changes in the chemical shifts of the remaining aromatic signals, due to the interruption of π-conjugation of the xanthene core.

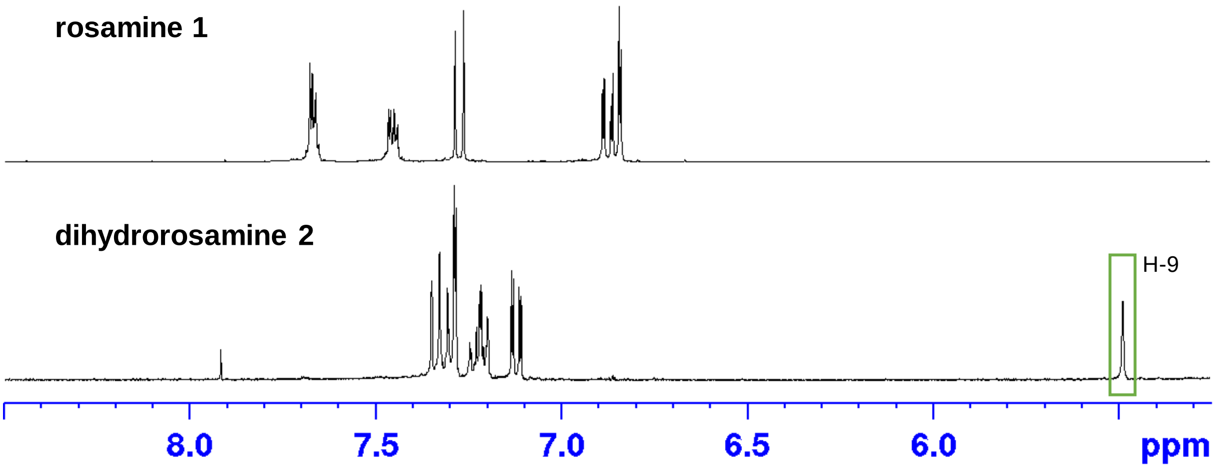

Figure S25. Comparison of the ^{1}H NMR spectra of **1** and **2**.

S9. Comparison of the ^{1}H NMR spectra of mono- and disubstituted derivatives **3** and **4**

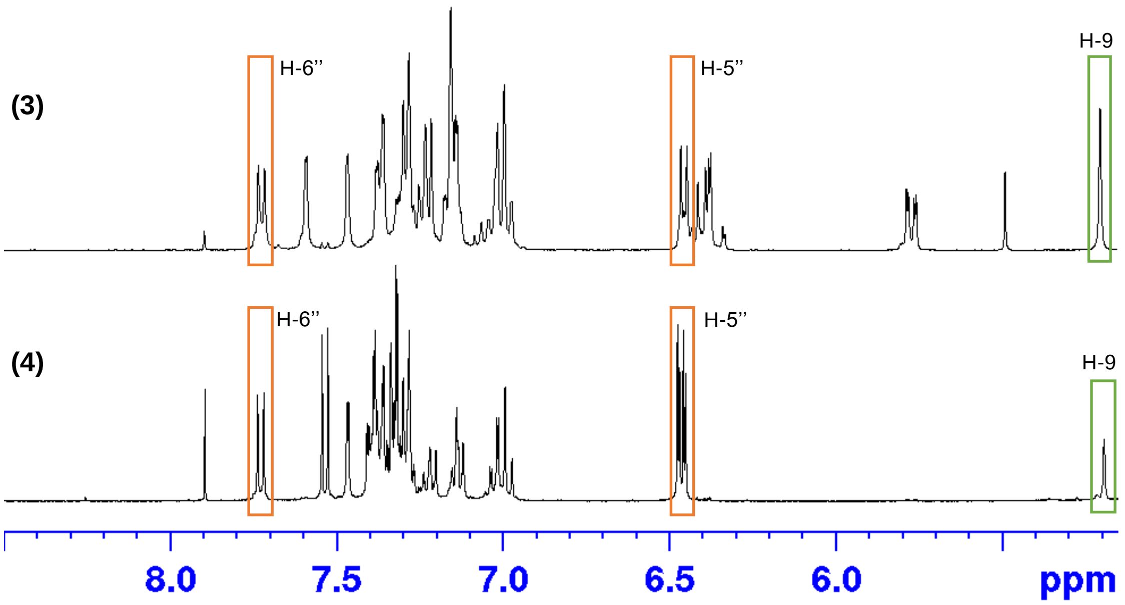

Figure S26. Aromatic region of the ^{1}H NMR spectrum of mono- and disubstituted derivatives **3** and **4**, in CD_3OD.

S10. Comparison of the ^{1}H NMR spectra of mono- and disubstituted derivatives **5** and **6**

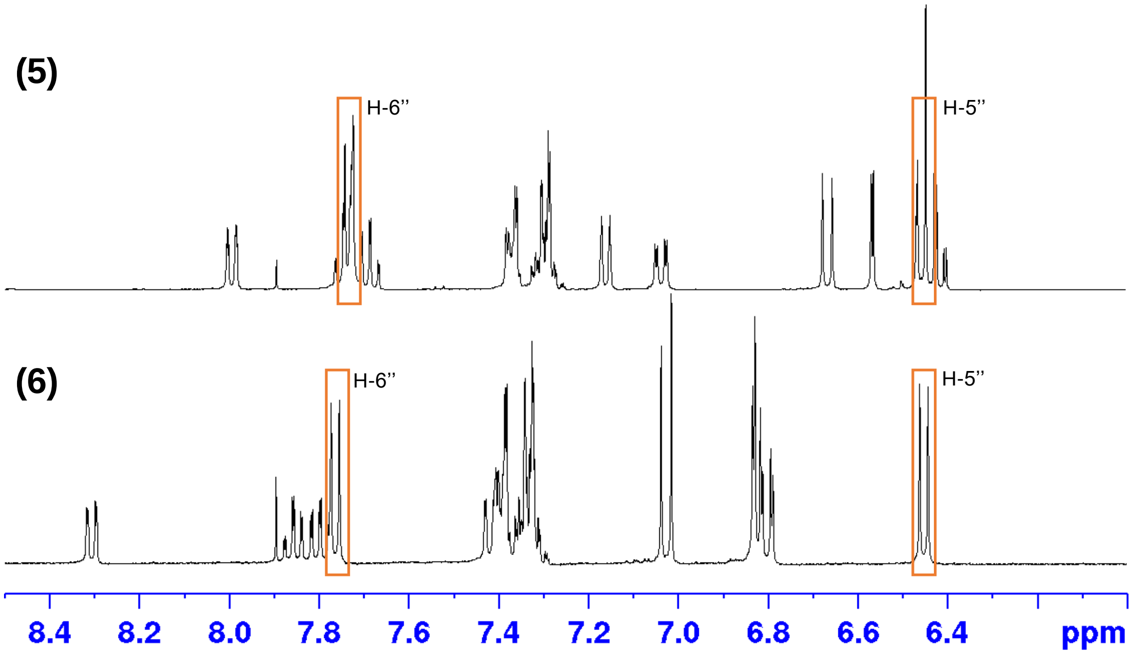

Figure S27. Aromatic region of the ^{1}H NMR spectrum of mono- and disubstituted derivatives **5** and **6**, in CD_3OD.

S11. MS spectra of rosamines and conjugates

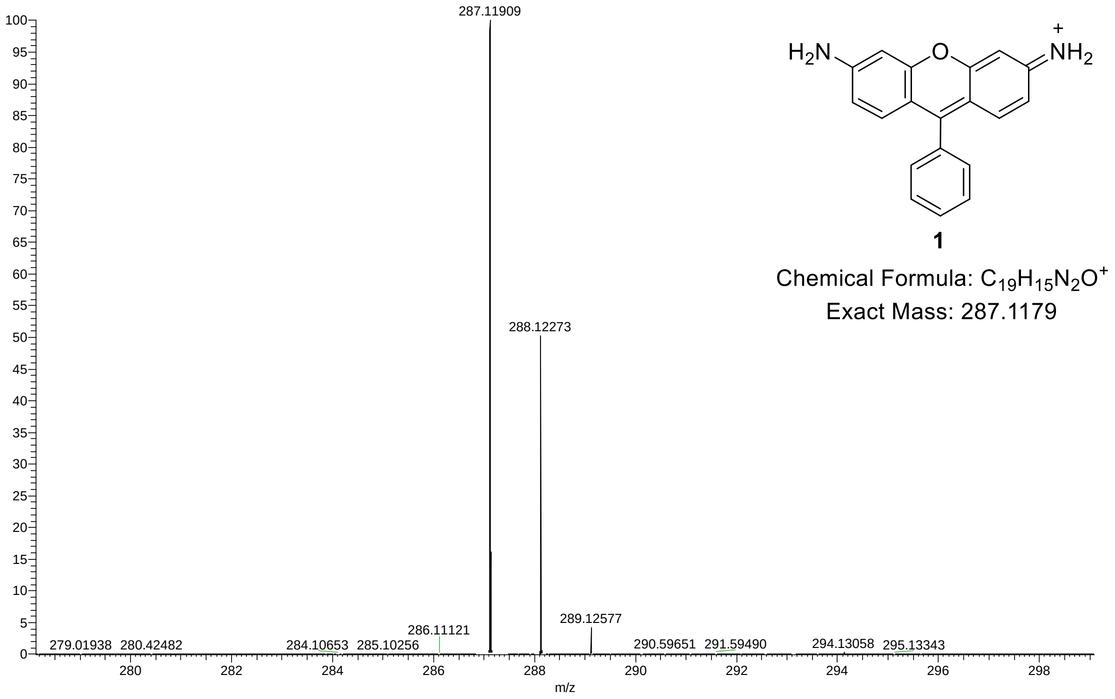

Figure S28. HRMS (ESI) of **1**.

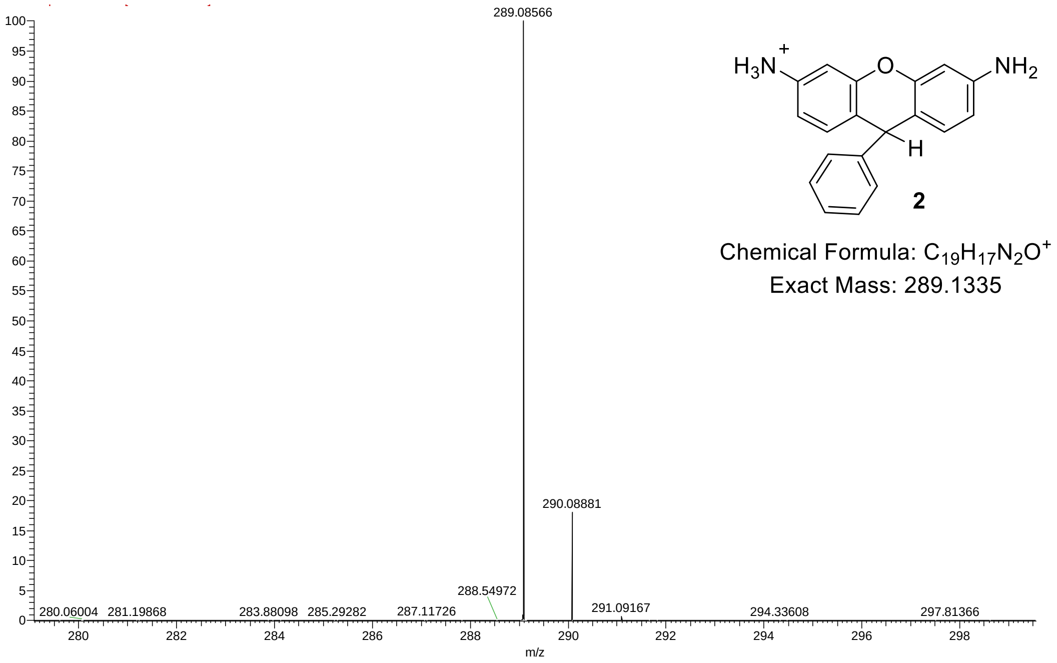

Figure S29. HRMS (ESI) of **2**.

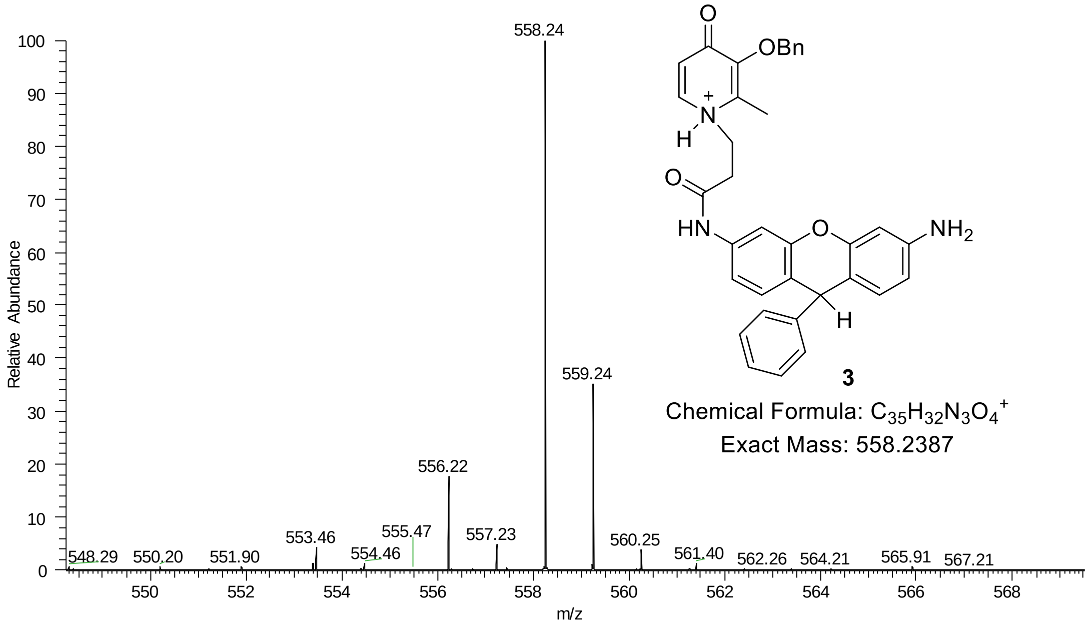

Figure S30. HRMS (ESI) of **3**.

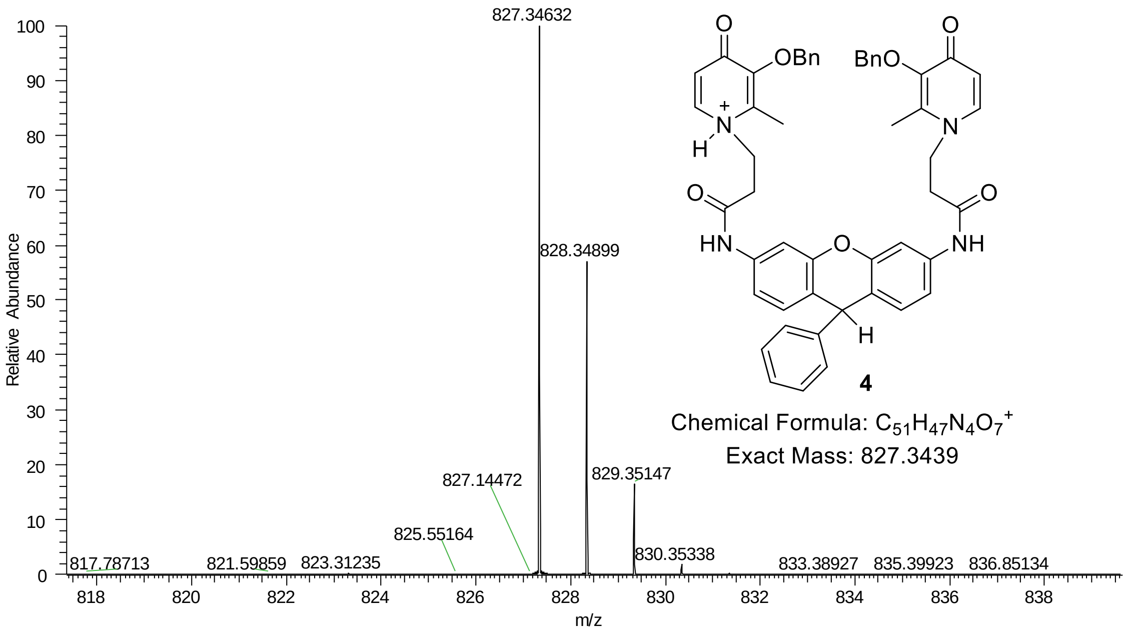

Figure S31. HRMS (ESI) of **4**.

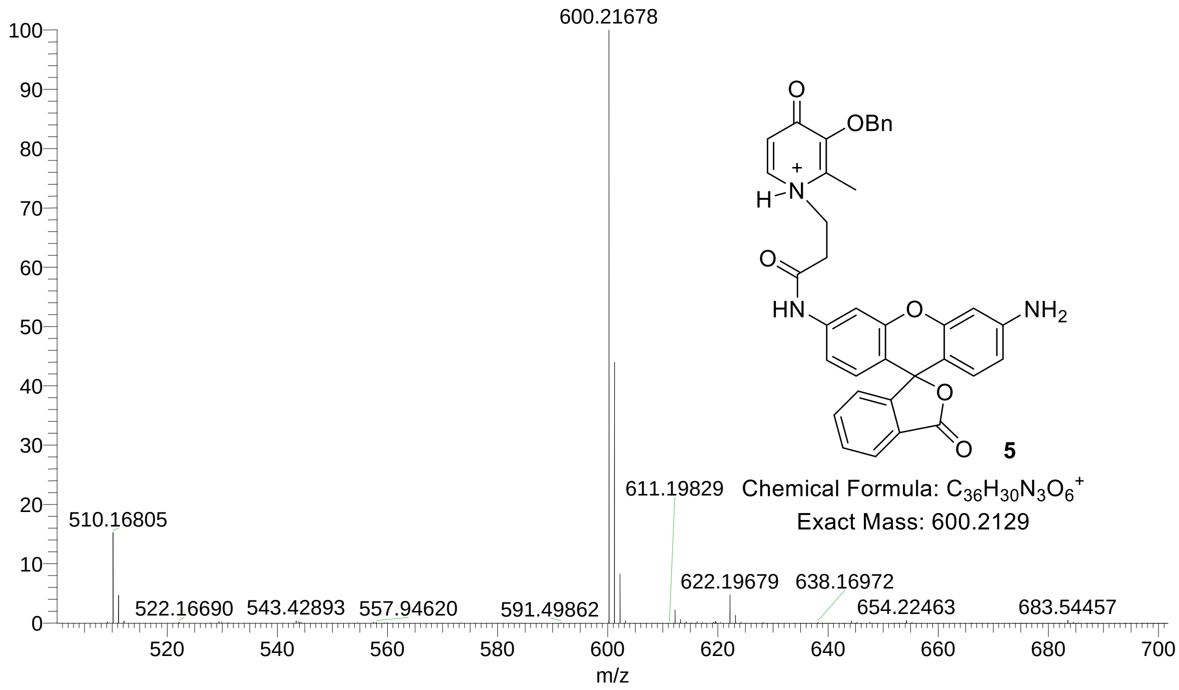

Figure S32. HRMS (ESI) of **5**.

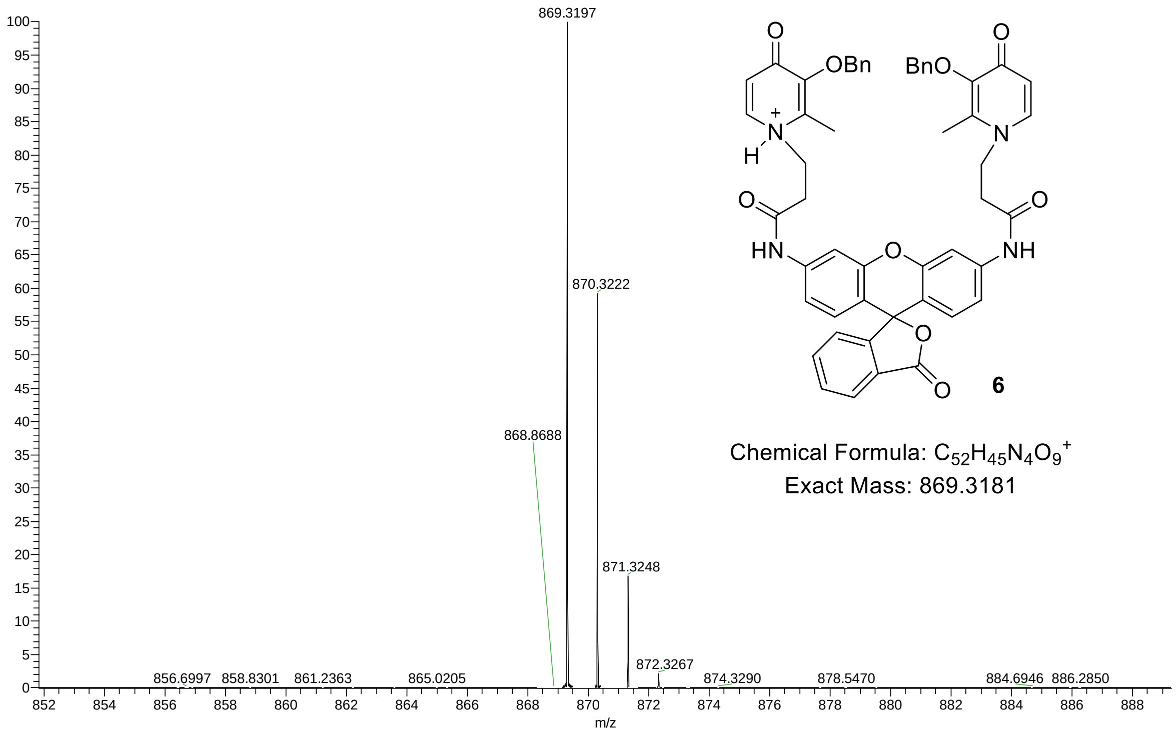

Figure S33. HRMS (ESI) of **6**.

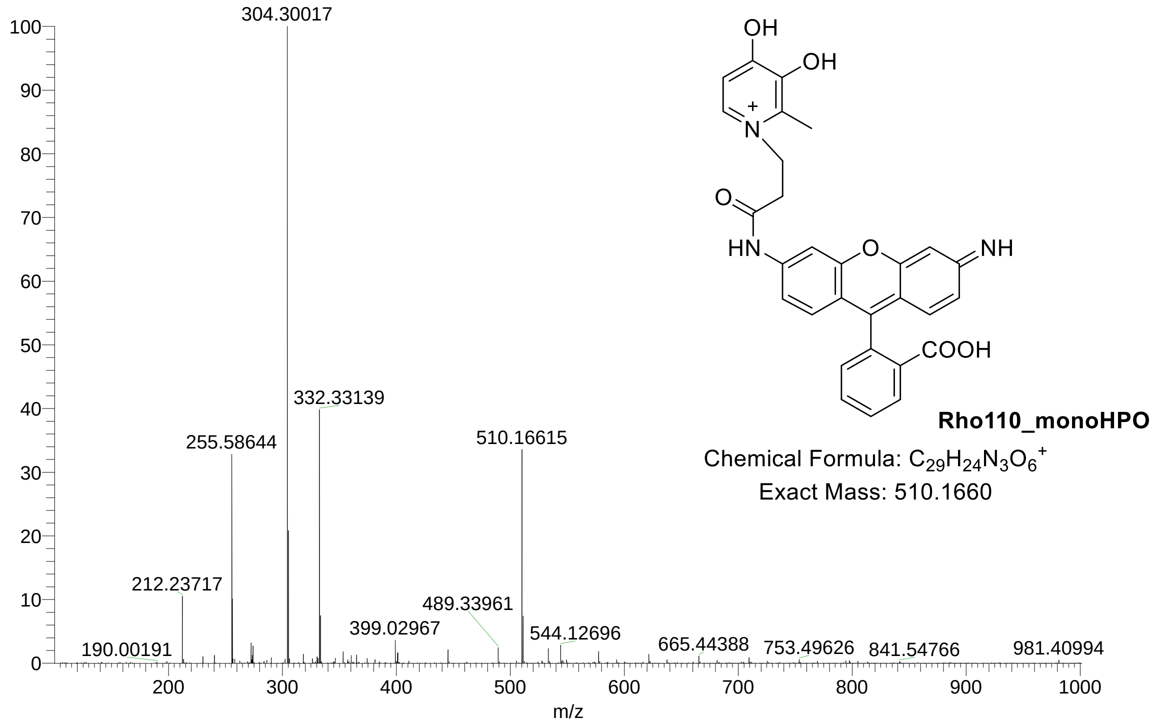

Figure S34. HRMS (ESI) of **Rho110-monoHPO**.

S12. Comparative absorption spectra of **Rho110**, **1**, **2**, **3** and **4**

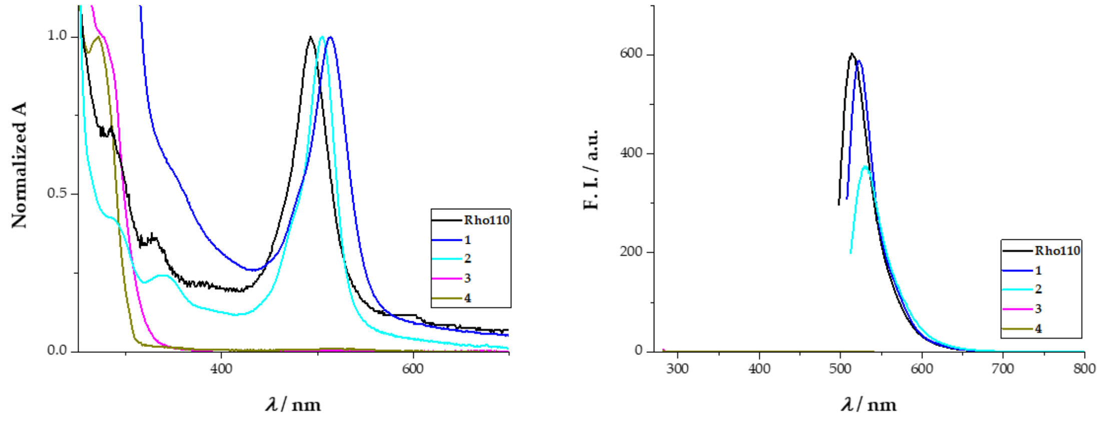

Figure S35. Absorption (normalized) and emission spectra of **Rho110**, **1–4** in CH_2Cl_2 at 25 ºC.

S13. Comparative absorption and emission spectra of spirolactone and quinoid forms of **5** and **6**

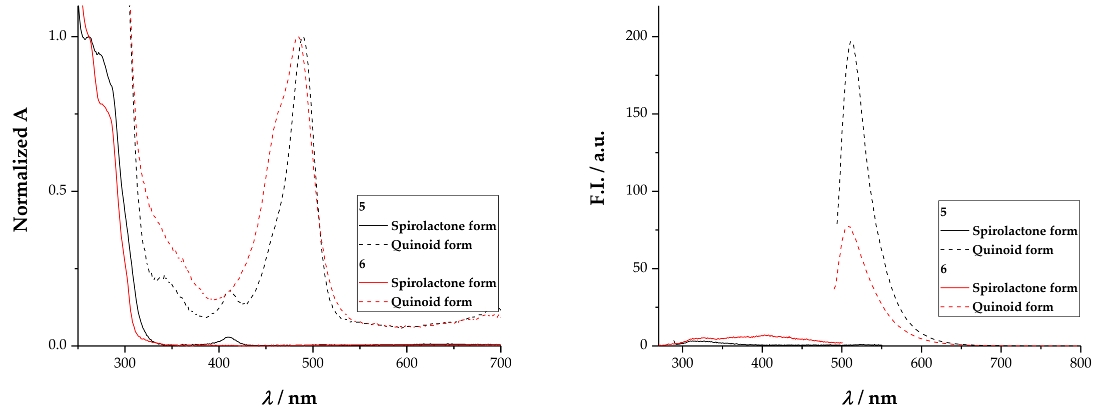

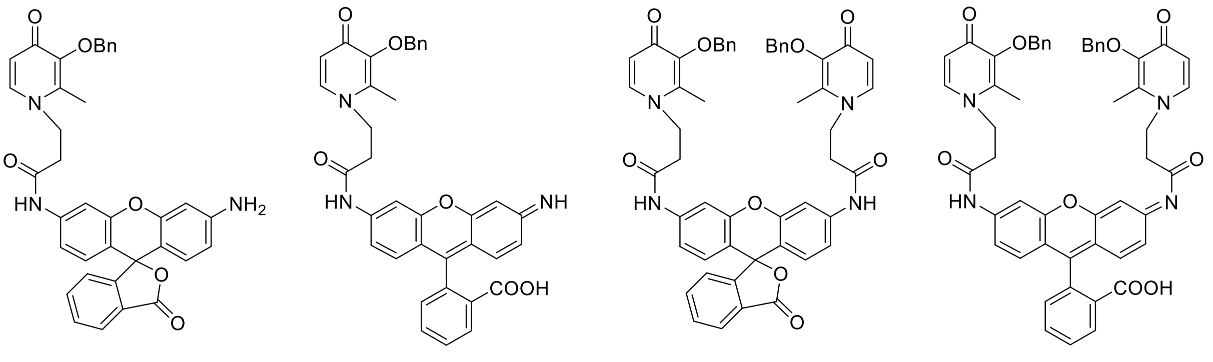

5 (spirolactone form) **5 (quinoid form)** **6 (spirolactone form)** **6 (quinoid form)**

Figure S36. Absorption and emission spectra of **5** and **6** in CH_2Cl_2 before (spirolactone form, full lines) and after an aliquot addition of HCl (37%) (quinoid form, dashed lines) at 25 ºC.
